# Supplementary material for: Cold spray as a powder metallurgy process for production of nickel aluminium bronze
Source: PLoS One. 2025 Mar 4;20(3):e0319333. doi: 10.1371/journal.pone.0319333 (PMC11878897; doi:10.1371/journal.pone.0319333)
Supplement: Attachment — Submitted filename: PONE-D-24-39008_R1_reviewer-2_comments.pdf [file pone.0319333.s004.pdf]

PLOS ONE

Cold spray as a powder metallurgy process for production of nickel aluminium bronze

--Manuscript Draft--

**Manuscript Number:**	PONE-D-24-39008R1
**Article Type:**	Research Article
**Full Title:**	Cold spray as a powder metallurgy process for production of nickel aluminium bronze
**Short Title:**	Additive manufacturing of nickel aluminium bronze
**Corresponding Author:**	Kannoorpatti Narayanan Krishnan, PhD<br>Charles Darwin University<br>Darwin, NT AUSTRALIA
**Keywords:**	Additive manufacturing, cold spray, nickel aluminium bronze, microstructure, powder metallurgy, HIP, tensile strength
**Abstract:**	Nickel aluminium bronze (NAB) alloys are known for their excellent strength and corrosion resistance, making them suitable for maritime and industrial applications. NAB is producible by Powder Metallurgy (PM) but typically requires high compaction pressure. The objective of this study is to investigate the manufacturing of NAB using the cold spray additive manufacturing (AM)process and to compare its properties to those produced by traditional methods such as casting and PM. Cold spray is a solid-state coating technique that accelerates powdered metal and carrier gas to supersonic speeds, enabling bonding through plastic deformation. Binary aluminium bronze (AB) and NAB alloys were produced using powders by cold spraying powders into 3D printed parts, and heat treating the resulting parts. The AB alloy contained blended 9.9% aluminium alloy (Al6061) powder and copper powder, while the NAB alloy included 11% Al6061 powder, 5.8% nickel powder, 6.8% iron powder, and copper powder. Powders were mixed under controlled conditions and deposited using a LightSPEE3D printer and compressed air. Post-deposition heat treatments, such as homogenisation, aging, and/or hot isostatic pressing (HIP), were applied to enhance material properties. The results indicate that the cold spray process, combined with appropriate heat treatments, can produce NAB alloys with desirable microstructures containing fine κ phases and mechanical properties with above 280 MPa yield strength, 500 MPa tensile strength and 20% elongation which are comparable to those achieved by traditional cast methods, and superior to PM methods. This study demonstrates the viability of cold spray AM to enhance the production of complex high-strength alloys, offering significant advancements for maritime and industrial applications.
**Order of Authors:**	Kannoorpatti Narayanan Krishnan, PhD
	Steven Camilleri
	Tien Tran
	Andrew Duguid
**Response to Reviewers:**	Thank you for your constructive feedback on the manuscript titled " Cold spray as a powder metallurgy process for production of nickel aluminium bronze." Your insightful comments have significantly improved the manuscript, and I am grateful for your guidance.<br>Address to reviewer’s comments<br>Reviewer #1:<br>First of all, the paper is quite attractive since it touchs a very ineresting topic, in particular related to this complexes systems, NAB. It should be modify in order to point out the relevance of microstrcuture and the relation with the process per se and the impact on mechanical properties.<br>The microstrcutural analysis is poor.<br>•We have added more information on microstructure analysis into the manuscript.<br><br>Figures 3 and 4 needs to identify better the phases and microprepitates. So, have K phases this new cold spray AM new NAB?. Figure 3, how did you identify this "beta phase"?. It looks like rather defects.

Powered by Editorial Manager® and ProduXion Manager® from Aries Systems Corporation

•All phases identified in the etched microstructure as example from the literature.

Where is the Cu in Future 2?. what are this black spot of Figure 2 b?.
•We have added Cu arrow into the figures 2b to highlight the location of Cu. The black spots are all porosity. We also added this information into the figure.
Fig 3 and Figure 4 perhaps need more time for the etching in order to reveal better the microstructure, since it is the ley point of your research.
•We have etched the samples again and replaced the old images.

You mention "better properties " of your cold sprayed AM NABm, in comparison traditional methods. It should be interesting point out the relevance of phases, percentages, average of the grain size, distribution and so on.
•Thank you for your valuable suggestions. Due to limited facilities, we are unable to precisely measure phase quantities and grain size. The term "better properties" in the manuscript refers primarily to the material's properties. We have revised this section in the manuscript to clarify and prevent any confusion.

The conclusions need to be reviewed in order to point out the real effect of microstructure.
•We have added more information in the conclusion section.
The references have some gramatical mistakes (ca ref 1).
•We have made correction.

Reviewer #2:
1.But cold spray also induces significant compressive force during deposition?
•Yes. It is noted.
2.AM?
•AM stands for Additive Manufacturing. We have made change in the manuscript.
3.“The results indicate that the cold spray process, combined with appropriate heat treatments, can produce NAB alloys with desirable microstructures and mechanical properties comparable to those achieved by traditional cast methods, and superior to PM methods.” – Examiner mentions, “This is a generic statement and does not provide any key information about the actual results of this study in both qualitative and quantitative values.”
•We have made change in the manuscript as follows:
“The results indicate that the cold spray process, combined with appropriate heat treatments, can produce NAB alloys with desirable microstructures containing fine κ phases and mechanical properties with above 280 MPa yield strength, 500 MPa tensile strength and 20% elongation which are comparable to those achieved by traditional cast methods, and superior to PM methods.”
4.Capitalised Cold Spray.
•We have made change in the manuscript.
5.“Nickel aluminium bronzes (NAB) are a popular set of alloys due to their excellent combination of properties. They have high strength and toughness, high wear, galling and cavitation resistances, are non-sparking, good corrosion and stress corrosion properties and biofouling resistance [1]. They are used extensively in aircraft landing gear bearings, marine propellers, pumps and valves, gears, and in non-sparking tools. They can be produced in cast, wrought or forged forms as well as by powder metallurgy. There are many different compositions of NAB alloy, but they generally contain up to 12% aluminium, 5% nickel, 7% iron, 3% manganese, and 2% silicon. The guide by Copper Development Association [1] is an excellent source of information on NAB. Aluminium is the primary element that strengthens the NAB alloys. Nickel and iron are added to improve corrosion and erosion resistance. Addition of iron also refines the structure and improves toughness. Manganese is added to improve fluidity of the alloys when casting. When nickel and iron, about 5%, are added to copper-aluminium alloy, two phases α and κ are produced. When NAB is quenched from 900oC, both α phase, martensite β and κ phases can form. On tempering at 500-715oC, the martensite phase transforms to α (Cu-rich) and eutectoid phase (κ and γ2) and secondary κ phases. The κ phase is found in 4 morphologies, locations and distributions. They are: KI – rosette form and iron-rich, κII – spheroidised form at”.
Examiner – “This information is primarily summarized from one source [1]. Please provide additional information from other published literature that can support these statements.”

•We have added more information along with references in the manuscript.
6.“Various kappa phases containing Ni and Fe are known to increase the mechanical properties of NAB. A decrease in elongation has been attributed to excess κ phases which form with an increase in aluminium content above nominal.” Examiner – “Please provide reference for this claim. Also mention the nominal aluminium content.”
•We have added more information along with references in the manuscript.
7.“WAAM” Examiner – “WAAM is mentioned in the previous paragraph as wire arc additive manufacturing.”
•We have made change in the manuscript.

8.“The first several paragraphs of the Introduction section was heavily focussed on microstructural phases exhibited in NAB alloys when subjected to different processing conditions. However, the later part of this section discussed more on additive manufacturing of NAB and the issues when printing using pre-alloyed powders. These two sections are quite distinct and there is no correlation drawn between microstructural variations in cast/forged/pht NAB parts with those of AMed NAB, as well as the mechanical property comparisions, which are the key results studied in this research work. Moreover, the Introduction lacks a pragraph on the research gaps and the importance/novelty of this study.
In my opinion, this section has to be considerably improved to better present the state-of-the-art literature on CSAM, its application to not only NAB but other related Cu-alloys.”
•We have made changes in the manuscript.
9.Why was Aluminium bronze studied in this work? The rationale behind this alloy addition should be discussed in the Introduction section.
•Aluminium bronze was selected to take advantages of using elemental blended with widely different melting temperatures.
10.What are the chemical compositions of the powders, their morphology, and their PSD? Was this the AL6061 alloy? What was the chemical composition of this powder? Is it 9.9% by weight or volume?
•Thank you for your insightful questions. We have revised the manuscript to include additional details about the powder, including its chemical composition. However, please note that the powder particle size distribution (PSD) is proprietary information, so we are unable to disclose it in the manuscript.
11.How long was the mixing carried out? Was it at room temperature? More experimental details are to be provided.
•We have made changes in the manuscript.
12.What was the powder feed rate?
•It is 60 g/min. We have made changes in the manuscript.
13.Were the same process parameters used to print both aluminium bronze and nickel aluminium bronze coupons?
•Yes. The two materials were deposited with the same parameters.
14.Does normal properties in this instance refers to cast or wrought mechanical properties?
•It refers to cast mechanical properties. We have changed the word normal to cast mechanical properties.
15.Why were these heat treatment temperatures selected? Can this heat treatment protocol be shown in the temperature schedule graph with respective heating and cooling rates? Also, was this HT followed for both AB and NAB?
•We have added the HT for the AB and a graph in the manuscript. Additionally, we have added an explanation for the temperature chosen for heat treatment.
16.Please present a table to show how many conditions were studied and mechanically tested in this work?
•Tables 1 and 2 provided the details.
17.For the XRD analysis, what was the x-ray source used? Also, the angular data capture rate?
•We have made changes in the manuscript as below:
The heat treated samples were studied using Xray diffraction (Malvern Panalytical) with copper X-ray target with the range from 25 to 90 degree at the rate of 4 degree per minute to study the phase components present.
18.There is no discussion of actual observations drawn from the micrographs shown in Figure 2.
•We have made changes in the manuscript.
19.How was density measured? This is not discussed in the Methodology section.

	•The density was measured by 100% - %porosity. We have added this in the methodology section.<br>20.Does this statement refer to similar composition or similar properties?<br>•The statement refers to similar density to PM. We have made changes to the statement to make it clearer.<br>21.This section is basically a discussion of published literature to compare relative densities achieved with that of PM process for various materials compared to that of cold spray process, which is relevant for an Introduction section. In this section, please discuss as to why the relative density of your NAB coupons in the as-sprayed condition is significantly higher than that reported in literature for other Cu-based cold sprayed parts. Is it owing to the nozzle design or process parameters or powder characteristics. A theory should be proposed and validated with subsequent experimentation. A recent study showed that the porosity percentage increases in heat treated aluminium bronze parts when compared with as-sprayed condition.<br>https://doi.org/10.1016/j.nxmate.2024.100312<br>•It is still unclear as to why we got high density for the materials. It could be attributed to the nozzle design, process parameters and powder characteristics. We have not use any other machine so we are unable to comment on comparing the density with others.<br>22.Why? Is it only for AB or NAB parts? More discussion of results is required in this section.<br>•This statement refers to both materials.<br>23.The first two paragraphs of this section should be discussed after presenting the results of this study. So, Fig. 3 should be presented and the key observations discussed, followed by literature based discussions.<br>•We have made changes to the manuscript.<br>24.How were the phases identified and correlated in the microstructure? Is there any EDS carried out to validate the statements made here.<br>•The phases were identified by comparing with phases from literature.<br>25.Since 5 coupons were tensile tested at each condition, the standard deviation should be mentioned for all mean values shown in Table 1.<br>•We have added this information.<br>26.Why was only sintering carried out for AB? Why not aging treatment?<br>•There is no aging behaviour in AB system with 9.9%Al, thus no aging treatment was needed.<br><br>27.These properties should also be presented in Table 1 along with the mechanical properties of similar parts produced by CSAM for better comparision. Table 2 should be merged with Table 1 for better comparision.<br>•These properties in different section, thus it is unsuitable to move the table to the previous section.<br>28.The Abstract mentions that the mechanical properties of cold sprayed NAB parts outperformed those of cast and PM parts. Where is this information presented in the Results and Discussion sections?<br>•The statement was in section 3.3 as follows:<br>“Table 2 suggests the mechanical properties of hipped material exceed the ASTM specification for cast Nickel aluminium bronze (C95800).”
**Additional Information:**	
**Question**	**Response**
**Financial Disclosure**<br><br>Enter a financial disclosure statement that describes the sources of funding for the work included in this submission. Review the submission guidelines for detailed requirements. View published research articles from *PLOS ONE* for specific examples.<br><br>This statement is required for submission	Yes

and **will appear in the published article** if the submission is accepted. Please make sure it is accurate.<br><br>**Funded studies**<br>Enter a statement with the following details:<br>• Initials of the authors who received each award<br>• Grant numbers awarded to each author<br>• The full name of each funder<br>• URL of each funder website<br>• Did the sponsors or funders play any role in the study design, data collection and analysis, decision to publish, or preparation of the manuscript?<br><br>Did you receive funding for this work?	
Please add funding details.<br>as follow-up to "**Financial Disclosure**<br><br>Enter a financial disclosure statement that describes the sources of funding for the work included in this submission. Review the submission guidelines for detailed requirements. View published research articles from *PLOS ONE* for specific examples.<br><br>This statement is required for submission and **will appear in the published article** if the submission is accepted. Please make sure it is accurate.<br><br>**Funded studies**<br>Enter a statement with the following details:<br>• Initials of the authors who received each award<br>• Grant numbers awarded to each author<br>• The full name of each funder<br>• URL of each funder website<br>• Did the sponsors or funders play any role in the study design, data collection and analysis, decision to publish, or preparation of the manuscript?<br><br>Did you receive funding for this work?"	This research was supported (partially or fully) by the Australian Government through the Department of Education's National Industry PhD Program (NIPhD 35017). The views expressed herein are those of the authors and are not necessarily those of the Australian Government or the Department of Education.<br>The work was partly funded by the generous support of Penn State ARL and SPEE3D. There was no additional external funding received for this study." in your updated Funding Statement.<br>The funder provided support in the form of salaries for authors TT, SC and AD, but did not have any additional role in the study design, data collection and analysis, decision to publish, or preparation of the manuscript. The specific roles of these authors are articulated in the 'author contributions' section.

Powered by Editorial Manager® and ProduXion Manager® from Aries Systems Corporation

Please select the country of your main research funder (please select carefully as in some cases this is used in fee calculation).<br>as follow-up to "**Financial Disclosure**<br><br>Enter a financial disclosure statement that describes the sources of funding for the work included in this submission. Review the submission guidelines for detailed requirements. View published research articles from *PLOS ONE* for specific examples.<br><br>This statement is required for submission and **will appear in the published article** if the submission is accepted. Please make sure it is accurate.<br><br>**Funded studies**<br>Enter a statement with the following details:<br>• Initials of the authors who received each award<br>• Grant numbers awarded to each author<br>• The full name of each funder<br>• URL of each funder website<br>• Did the sponsors or funders play any role in the study design, data collection and analysis, decision to publish, or preparation of the manuscript?<br><br>Did you receive funding for this work?"	AUSTRALIA - AU
**Competing Interests**<br><br>Use the instructions below to enter a competing interest statement for this submission. On behalf of all authors, disclose any competing interests that could be perceived to bias this work—acknowledging all financial support and any other relevant financial or non-financial competing interests.<br><br>This statement is required for submission and **will appear in the published article** if the submission is accepted. Please make sure it is accurate and that any funding	I have read the journal's policy and the authors of this manuscript have the following competing interests:<br>Steven Camilleri, Tien Tran and Andrew Duguid are employees of SPEE3D. This has been declared in the authorship details.

Powered by Editorial Manager® and ProduXion Manager® from Aries Systems Corporation

sources listed in your Funding Information later in the submission form are also declared in your Financial Disclosure statement.<br><br>View published research articles from *PLOS ONE* for specific examples.<br><br>**NO authors have competing interests**<br><br>Enter: *The authors have declared that no competing interests exist.*<br><br>**Authors with competing interests**<br><br>Enter competing interest details beginning with this statement:<br><br>*I have read the journal's policy and the authors of this manuscript have the following competing interests: [insert competing interests here]*<br><br>* typeset	
**Ethics Statement**<br><br>Enter an ethics statement for this submission. This statement is required if the study involved:<br><br>• Human participants<br>• Human specimens or tissue<br>• Vertebrate animals or cephalopods<br>• Vertebrate embryos or tissues<br>• Field research<br><br>Write "N/A" if the submission does not require an ethics statement.<br><br>General guidance is provided below. Consult the submission guidelines for detailed instructions. **Make sure that all information entered here is included in the Methods section of the manuscript.**	N/A

Powered by Editorial Manager® and ProduXion Manager® from Aries Systems Corporation

**Format for specific study types**<br><br>**Human Subject Research (involving human participants and/or tissue)**<br>• Give the name of the institutional review board or ethics committee that approved the study<br>• Include the approval number and/or a statement indicating approval of this research<br>• Indicate the form of consent obtained (written/oral) or the reason that consent was not obtained (e.g. the data were analyzed anonymously)<br><br>**Animal Research (involving vertebrate animals, embryos or tissues)**<br>• Provide the name of the Institutional Animal Care and Use Committee (IACUC) or other relevant ethics board that reviewed the study protocol, and indicate whether they approved this research or granted a formal waiver of ethical approval<br>• Include an approval number if one was obtained<br>• If the study involved *non-human primates*, add *additional details* about animal welfare and steps taken to ameliorate suffering<br>• If anesthesia, euthanasia, or any kind of animal sacrifice is part of the study, include briefly which substances and/or methods were applied<br><br>**Field Research**<br><br>Include the following details if this study involves the collection of plant, animal, or other materials from a natural setting:<br>• Field permit number<br>• Name of the institution or relevant body that granted permission	
**Data Availability**<br><br>Authors are required to make all data underlying the findings described fully available, without restriction, and from the time of publication. PLOS allows rare exceptions to address legal and ethical concerns. See the PLOS Data Policy and FAQ for detailed information.	Yes - all data are fully available without restriction

A Data Availability Statement describing where the data can be found is required at submission. Your answers to this question constitute the Data Availability Statement and **will be published in the article**, if accepted.<br><br>**Important:** Stating 'data available on request from the author' is not sufficient. If your data are only available upon request, select 'No' for the first question and explain your exceptional situation in the text box.<br><br>Do the authors confirm that all data underlying the findings described in their manuscript are fully available without restriction?	
**Describe where the data may be found in full sentences. If you are copying our sample text, replace any instances of XXX with the appropriate details.**<br><br>• If the data are **held or will be held in a public repository**, include URLs, accession numbers or DOIs. If this information will only be available after acceptance, indicate this by ticking the box below. For example: *All XXX files are available from the XXX database (accession number(s) XXX, XXX.*).<br>• If the data are all contained **within the manuscript and/or Supporting Information files**, enter the following: *All relevant data are within the manuscript and its Supporting Information files.*<br>• If neither of these applies but you are able to provide **details of access elsewhere**, with or without limitations, please do so. For example:<br><br>*Data cannot be shared publicly because of [XXX]. Data are available from the XXX Institutional Data Access / Ethics Committee (contact via XXX) for researchers who meet the criteria for access to confidential data.*<br><br>*The data underlying the results presented in the study are available from (include the name of the third party*	All relevant data are within the manuscript and its Supporting Information files.

and contact information or URL).<br>• This text is appropriate if the data are owned by a third party and authors do not have permission to share the data.<br><br>* typeset	
Additional data availability information:	

Powered by Editorial Manager® and ProduXion Manager® from Aries Systems Corporation

Manuscript Click here to access/download;Manuscript;Manuscript.docx

Cold spray as a powder metallurgy process for production of nickel aluminium bronze

Steven Camilleri [1,2¶], Tien T T Tran [1,2¶], Andrew Duguid [2¶], Krishnan Kannoorpatti [1*]

[1] Advanced Manufacturing Alliance, Energy and Resources Institute, Charles Darwin University, Darwin, Australia

[2] SPEE3D, Charles Darwin University, Australia

*Corresponding author

E-mail: Krishnan.kannoorpatti@cdu.edu.au (KK)

Abstract

Nickel aluminium bronze (NAB) alloys are known for their excellent strength and corrosion resistance, making them suitable for maritime and industrial applications. NAB is producible by Powder Metallurgy (PM) but typically requires high compaction pressure. The objective of this study is to investigate the manufacturing of NAB using the cold spray additive manufacturing (AM)process and to compare its properties to those produced by traditional methods such as casting and PM. Cold spray is a solid-state coating technique that accelerates powdered metal and carrier gas to supersonic speeds, enabling bonding through plastic deformation. Binary aluminium bronze (AB) and NAB alloys were produced using powders by cold spraying powders into 3D printed parts, and heat treating the resulting parts. The AB alloy contained blended 9.9% aluminium alloy (Al6061) powder and copper powder, while the NAB alloy included 11% Al6061 powder, 5.8% nickel powder, 6.8% iron powder, and copper powder. Powders were mixed under controlled conditions and deposited using a LightSPEE3D printer and compressed air. Post-deposition heat treatments, such as homogenisation, aging, and/or hot isostatic pressing (HIP), were applied to enhance material properties. The results indicate that the cold spray process, combined with appropriate heat treatments, can produce NAB alloys with desirable microstructures containing fine κ phases and mechanical properties with above 280 MPa yield strength, 500 MPa tensile strength and 20% elongation which are comparable to those achieved by traditional cast methods, and superior to PM methods. This study demonstrates the viability of cold spray AM to enhance the production of complex high-strength alloys, offering significant advancements for maritime and industrial applications.

Introduction

Metal manufacturing has evolved significantly over the centuries, transitioning from traditional methods like casting and forging to more sophisticated techniques such as sheet metal forming and powder metallurgy (PM). In recent decades, additive manufacturing (AM) of metal has emerged, becoming increasingly commercially viable and reliable, particularly over the last ten years.

Among these innovative methods, the cold spray technique, also known as gas dynamic cold spray, stands out as a uniquely solid-state coating process that propels powdered metal and a carrier gas to supersonic speeds through a de Laval nozzle. Unlike traditional thermal spray techniques, which often lead to significant thermal degradation, oxidation, or phase transformations, cold spray ensures that upon impact with a substrate, the particles undergo plastic deformation and bond to the surface or prior particles without significant heating. While AM is lauded for its ability to create objects with customisable geometries without the need for traditional tooling, it is less recognized for its ability to customise material properties. By mixing elemental metal powders rather than using prealloyed powders, various alloys can be created providing greater flexibility. This flexibility leads us to compare cold spray additive manufacturing with powder metallurgy. In this paper, the production of nickel aluminium bronze using cold spray method is compared with powder metallurgy process.

Nickel aluminium bronzes (NAB) are a popular set of alloys due to their excellent combination of properties. They have high strength and toughness, high wear, galling and cavitation resistances, are non-sparking, good corrosion and stress corrosion properties and biofouling resistance [1]. They are used extensively in aircraft landing gear bearings, marine propellers, pumps and valves, gears, and in non-sparking tools. They can be produced in cast, wrought or forged forms as well as by powder metallurgy. There are many different compositions of NAB alloy, but they generally contain up to 12% aluminium, 5% nickel, 7% iron, 3% manganese, and 2% silicon. The guide by Copper Development Association [1] is an excellent source of information on NAB. Aluminium is the primary element that strengthens the NAB alloys. Nickel and iron are added to improve corrosion and erosion resistance. Addition of iron also refines the structure and improves toughness. Manganese is added to improve fluidity of the alloys when casting.

When nickel and iron, about 5%, are added to copper-aluminium alloy, two phases α and κ are produced. When NAB is quenched from 900°C, both α phase, martensite β and κ phases can form. On tempering at 500-715°C, the martensite phase transforms to α (Cu-rich) and eutectoid phase (κ and γ_2) and secondary κ phases. The κ phase is found in 4 morphologies, locations and distributions. They are: K_I – rosette form and iron-rich, κ_{II} – spheroidised form at

the grain boundaries, κ_{III} – lath – shaped lamellar (Ni-rich) and κ_{IV} – fine precipitate inside grains and iron-rich. The precipitates being rich in certain elements tend to get selectively corroded [1] as these phases set up galvanic couples with the other phases.

The corrosion resistance of NAB has been attributed to the passive layers of oxides of aluminium and copper [2]. The passive layers also provide excellent resistance to flow induced corrosion due to the tenacity of the protective films.

Various kappa phases containing Ni and Fe are known to increase the mechanical properties of NAB. A decrease in elongation has been attributed to excess κ phases which form with an increase in aluminium content above nominal.

Iron more than 3 wt% reduces grain growth at high temperatures and produces a smaller grain size and solidification range. Addition of iron allows NAB to retain strength at high temperatures [3]. Nickel addition refines grains by retarding β phase formation. However, nickel content should not be lower than the iron content as this will lower corrosion resistance. A nominal content of 5 wt % Ni and 4 wt % Fe provides the optimal high strength along with corrosion resistance [3]. The properties of NAB are dependent on how well the phases are refined and distributed in the alloy. If the κ phases in which segregation of elements occurs can be reduced, then the properties will be more uniform [4].

This NAB alloy are produced mainly by casting processes. This can also be produced by PM processes. These can be fabricated using welding processes. Recently, the NAB alloys are being manufactured by AM methods. Different manufacturing processes produce different microstructures and properties based on processing history. Selected Laser Melting (SLM) fabricated NAB alloys produced uniform martensitic structures with nano-sized Fe-rich particles (κ_{IV}) [5]. Annealing transformed the microstructure into α and lamellar/spheroidized Ni-rich particles. Martensite and κ_{IV} particles were not fully transformed.

Many of the Wire Arc Additive Manufacturing (WAAM) processes described in previous work significantly alter the microstructure of NAB producing grain growth and martensite in the heat affected zone [6-8]. The fusion zone exhibited Widmanstätten α and very fine martensite [9]. Martensite is harder and brittle and susceptible to corrosion and cavitation damage. After a Post-Weld Heat Treatment (PWHT) at 675°C for six hours refined the martensite to α and κ phases. The WAAM parts generally had better or equal mechanical properties to that of cast materials. κ_{I} was not precipitated due to the rapid cooling rates [7, 9].

In additive manufacturing using beam processes, the powders used are generally alloy powders [8, 10]. With the use of alloy powders, there is no flexibility to vary or compensate the composition of the alloy to get desired properties. Similarly, using WAAM, the wires used are standard wires and to make changes to the composition would require a whole new wire

manufacturing run. Even powder metallurgy NAB products are manufactured using alloy powders [11, 12]. The lack of compositional flexibility can be addressed by using other processes that can produce the required NAB alloy from elemental powders. Chen et al [13] had provided a review of the use of elemental powders for laser powder directed energy deposition process. Some of the issues highlighted are that each elemental powder will reflect laser beam differently and their addition into the melt pool is inconsistent. This would require remelting of each layer a second time. In some cases, some of the elemental powders failed to melt and/or evaporated at different rates, leading to compositional error in the final alloy.

Cold spray AM is an emerging technique in AM that provides advantages for material deposition and alloy production. Cold spray AM operates as a solid-state process, using pressurised gas to accelerate metal powders at supersonic speeds onto a substrate. This allows the formation of a cohesive and dense layer without melting. Elemental powders can be blended and cold spayed and heat treated to produce complex alloys instead of using prealloyed powder. Cold spray AM is a suitable manufacturing method for powder with widely varying melting temperature, making it ideal for producing complex alloys with customised properties [14].

The use of elemental powders in cold spray AM provides opportunities for improving the production process and the properties of NAB alloys. This is because the process does not melt the material, so vastly differing melting temperatures of the elements do not present a concern. Additionally, the elemental powders are a lower cost than alloyed powders. Production of NAB alloys by cold spray AM has been attempted by Peng et al using NAB prealloyed powder [15]. It was found that the deposition efficiency was only 7.61% and it increased to 30.56% after annealing at the powder at 600°C for 10 hours. Heat treatment was done on the alloyed powder to modify the microstructure and to reduce the hardness. The deformation of hard, segregated alloy powder is less practical by cold spray unless a more expensive process relying on high pressure equipment or helium gas is used [16].

In this research, an attempt has been made to produce NAB which addresses some of the difficulties faced by other processes by using elemental powder, high pressure cold spray AM method and using air as the propellant.

Materials and methodology

Materials feed stock

Aluminium bronze was selected for cold spray AM to take advantages of using elemental blended with widely different melting temperatures. A binary aluminium bronze (AB) was

fabricated by using cold spray process with a mixed powder of elemental copper and aluminium alloy (Al6061). Separately a nickel aluminium bronze (NAB) alloy was fabricated by using cold spray process with a mixed powder comprised of elemental copper, aluminium alloy (Al6061), elemental nickel and elemental iron powders. The composition ratios for AB were 9.9 wt% aluminium alloy Al6061 and the remainder copper. For NAB, the ratios were 11 wt% aluminium alloy, 5.8 wt% nickel, 6.8 wt% iron, and the remainder copper. The powders used in this process were gas-atomised powder and were supplied by SPEE3D.

The powders were mixed by a V-blender powder mixing machine for 1.5 hours at room temperature. Argon gas was introduced during mixing to displace the air inside the chamber and reduce the formation of oxide during mixing. Powder moisture was controlled by adding silica gel desiccant into the powder mixer for further removing moisture.

Cold spray AM process

To form a cold spray compact, it is necessary to load the blended powder into the powder feeder of a cold spray AM apparatus, select a toolpath that will provide the right size and shape tensile specimen, set the cold spray process parameters, and then form the block. The cold spray AM apparatus chosen was a LightSPEE3D printer from the Australian company SPEE3D, equipped with their newer Phaser cold spray nozzle. The process gas was chosen to be compressed air in order to minimise process cost. The machine holds the cold-spray nozzle in a fixed position while a robot arm moves the workpiece over the particle-laden jet according to a deposition strategy determined by a proprietary 3D slicing algorithm. The carrier gas, air, is compressed to 30 bar, heated to 540°C with 60 g/min. The standoff distance from the nozzle head to the build surface was fixed at 20 mm. Printed parts for tensile testing were rectangle blocks with dimensions of 20 mm x 32 mm x 106 mm in size. After forming the parts were subjected to different heat treatments and machined into groups of 5 tensile samples using EDM wire cutting (Fig 1) according to ASTM E8.

Fig 1. Tensile sample design

Heat treatment process and testing

Press and sinter powder metallurgy relies on a wide range of sintering heat treatments to properly process a wide range of material systems. Advanced production PM furnaces are able to heat treat in hydrogen, or disassociated ammonia, or even inert atmospheres. In our case the sintering was carried out using an atmospheric furnace heat treatment (i.e., with minimum sintering cost) and in a separate experiment, using hot isostatic pressing using argon gas. This was done so a determination could readily be made if it was possible to obtain standardproperties for the material system with casting.

Fig 2. Heat treatment profile for each material

The as-sprayed AB parts were homogenised at 1000°C for 14 hours and furnace cool to 900°C for 2.5 hours and quench in soluble oil. The as-sprayed NAB parts were homogenised at 1000°C for 14 hrs, increase temperature to 1035°C for 2.5 hours and furnace cool to 900°C for 1 hours and quench in soluble oil then followed by aging at 625°C for 2 hrs followed by quenching in soluble oil (Fig 2). With inclusion of HIP (Hot Isotactic Pressing) process, after the homogenisation step at 1000°C, the parts were then HIPed at 1035°C and 1000 bar for a 5-hour dwell, followed by a solution treatment at 1000°C to dissolve precipitates caused by the slow cooling process after HIPing. The parts were then aged at 625°C for 2hrs. The temperatures were chosen to produce sintering and homogeneous alloy based on previous literatures on PM on a similar alloy [17].

Metallography and X-ray Diffraction

The specimens were polished to 0.6μm finish and etched with Carpenter etchant ($FeCl_3$, $CuCl_2$, Hydrochloric acid Nitric acid and Ethanol). Microscopic observations were done using Nikon 600 optical microscope. Porosity of the as-sprayed samples were measured using imageJ analysis [18, 19]. The heat treated samples were studied using Xray diffraction (Malvern Panalytical) with copper X-ray target with the range from 25 to 90 degree at the rate of 4 degree per minute to study the phase components present.

Results and discussion

Relative density

Fig 3. Microstructure of as-sprayed alloy (a) Binary Aluminium Bronze (b) Nickel Aluminium Bronze

Fig 3 shows the respective elements present in the right macro concentration as a metal matrix composite. The porosity was measured to be about 2% (S1 Data) in the material which means that the density was about 98%. Visibly, aluminium distributed uniformly through the as-sprayed samples. In contrast, literature on the PM method shows the density reaches only 88% - 89.3% of a mixed powder of Cu-12Al-xNi which were compacted at 550 MPa and sintered at 1000°C [17]. Additionally, Cu-10Al-xAg also exhibit about 69% - 76% relative density with compact pressure at 200 MPa and sintered at 850°C [20]. This suggests the capability of cold spray AM to produce similar compacts density to PM but in higher density without the need for such high pressures.

It is known that traditional PM has ability to create high-density materials, typically in the range of 75% to 98% of the theoretical density, however this comes with significant limitations. The density and porosity of a product produced by the PM method is regulated by compaction pressure. The higher the compaction pressure, the greater the density, the lower the porosity, and the greater the final mechanical properties [21]. Mechanical, electrical, and other properties of a material are dependent on the density and porosity, which assumes, therefore, their indirect dependence on compaction pressure. Achieving densities above 90% typically requires very high compaction pressures (600-700 MPa) for aluminium – based material [22]. Additionally, the literature reports that iron and low-alloy steel powders require a compacted pressures at 600 MPa to 800 MPa to attain green densities of 85% to 90% of the pore-free solid density [23, 24]. Copper powders compacted at 600 MPa exhibit green densities of 88-93% of the theoretical density [23]. Mahani et al. examined the impact of compaction pressure on the hardness of Cu–W-C (graphite) alloy fabricated by powder metallurgy [25]. By increasing the compaction pressure, hardness increased due to a reduction in the porosity of the material.

In comparison, cold sprayed AM and coatings have also demonstrated high green densities which do not require high pressure compact process. Aluminium and aluminium alloy coatings have been shown to possess green densities in the range of 90-98% of the theoretical density [26]. Ningsong et. al. [27] indicated that the density of pure aluminium parts produced by cold spray can reach up to 99.93%. For titanium alloy Ti6Al4V, reported densities range from 70% to 99%: Vo et. al. reported 93% density with spraying in N_2 [28]; Wong et. al. reported 77.6% density with spraying in air [29]. Pure copper coatings have been reported to exhibit a density of at least 98% [30]. Cold spray AM of Amstrong process Titanium powder also exhibit as high density as PM process using the same powder pressed at above 700 MPa [31, 32]. Fully dense titanium parts were fabricated using the cold spray AM process. This was accomplished using low-pressure and low-temperature nitrogen as the process gas. The key to this result is attributed to the unique coral-shaped powder morphology that accelerates well in the gas flow, allowing the easy densification and deposition through cold spray [32].

In powder metallurgy, it is common practice to use blended elemental powders or a mixture of a base metal powder and master alloy powders, rather than employing pre-alloyed powders of the desired composition [33]. This approach offers several advantages: it is more cost-effective than relying completely on pre-alloyed powders, it enables tailoring of properties such as particle size distribution and flowability, it facilitates better control of interdiffusion and homogeneity during sintering compared to pre-alloyed powders, and it provides flexibility in alloy design by allowing the production of compositions that may be difficult or impossible to obtain via melting and atomization of pre-alloyed powders. With blended powders, powder

metallurgy has demonstrated high densities. Our study with cold spray AM also demonstrated high densities.

Microstructural characteristics

Fig 4. After Heat treatment, microstructure of (a-b) Binary Aluminium Bronze; (c) Nickel Aluminium Bronze

Phases were identified according to literature review on microstructure of NAB [34]. The microstructural evolution and phase transformations observed in aluminium bronze and nickel aluminium bronze after heat treatment are illustrated in Fig 4. Binary aluminium bronze shows the presence of both alpha phase as a major phase and beta phase (Figs 4 a and b). It is evident that the materials have undergone homogenization, resulting in the formation of well-defined alloy phases. The nickel aluminium bronze exhibits a predominantly single alpha phase microstructure with fine precipitates (Fig 4 c). The formation of these precipitates obtained by the aging process contributes to an increase in the material's tensile strength by means of precipitation hardening mechanisms. These microstructural observations highlight the efficacy of the heat treatment process in promoting interdiffusion, phase transformations, and the development of tailored microstructures, ultimately enhancing the mechanical properties of the respective aluminium bronze and nickel aluminium bronze alloys.

The heat treatment (HT)/sintering process is a crucial step in powder metallurgy involving mixed or blended powders, such as elemental powders or a combination of a base metal powder and master alloy powders. This thermal treatment facilitates interdiffusion and homogenization, enabling the formation of the desired alloy composition, phase constituents, and microstructure. The extent of diffusion bonding and grain growth depends on the sintering temperature and time. Higher temperatures improve the bonding between the particles and, hence, increases the strength of a material [22, 25] and densification [24]. Without proper heat treatment, the sintered components fabricated from mixed powders would exhibit incomplete alloying, high porosity, inhomogeneous microstructure, and inferior mechanical performance.

A similar phenomenon is observed in the cold spray process. The literature has demonstrated that heat treatment of cold-sprayed mixed powders, such as Cu-Ni, can help reduce the as-sprayed porosity and facilitate the formation of a homogenised alloy [35]. Literature also suggests that the strength of cold spray AM Aluminium alloy deposits can be increased by introducing small precipitates through performing heat treatment (HT) in age hardenable alloys, such as 2xxx, 6xxx, and 7xxx Al [36-38]. This is due to the fact that deformation before heat treatment is often used to promote non-uniform nucleation of precipitates in these alloys. Other CS copper and copper alloys are also reported to have better mechanical performance

after HT [39-42]. Likewise, the current study shows cold spray AM aluminium bronze and nickel aluminium bronze can form homogenized alloys through a simple heat treatment process in a low cost air atmosphere.

Mechanical properties and further enhancing mechanical properties with the HIP process

To optimise the properties of the material, heat treatment processes were developed. The material properties post heat treatment is listed in Table 1. It should be noted that a constraint for total heat treatment time was introduced deliberately so that parts could be completely manufactured within a 24-hour cycle, including part printing, heat treatment etc. Consequently, the AB heat treatment was limited to 16.5 hours and the NAB heat treatment was limited to 18 hours. There was good evidence of the potential to improve the properties even further with longer heat treatments.

Table 1 Material properties with heat treatment only – no HIP

	**Process**	**Average Yield Strength (MPa)**	**Average Ultimate Tensile Strength (MPa)**	**Average Elongation (%)**	**Average Hardness (Brinell)**
**Binary Aluminium Bronze**	Sintering	160 ± 1.5	300 ± 2.9	8 ± 0.6	87 ± 1.7
**Nickel Aluminium Bronze**	Sintering and aging	257 ± 1.1	404 ± 2.9	10 ± 0.7	150 ± 1.9

The addition of alloying elements, such as Fe and Ni, plays a crucial role in enhancing the overall properties of the materials under consideration [17]. A higher concentration of these alloying elements contributes to an increase in tensile strength; however, it is accompanied by a reduction in elongation. Interestingly, despite the cost-effective approach adopted for heat treatment, the resulting material properties approach the properties specified by ASTM B505 cast nickel aluminium bronze standard.

Furthermore, other cold-sprayed compacts were subjected to atmospheric furnace heat treatments along with Hot Isostatic Pressing (HIP), leading to the microstructures shown in

Fig 5. It is evident that the inclusion of a HIP process has resulted in reduced porosity and improved homogenization within the material.

Fig 5. Microstructure of HIPed Aluminium Bronze

Fig 6. XRD of HIPed Nickel Aluminium Bronze

The XRD analysis, shown in Fig 6, of the NAB produced by cold spray AM revealed patterns that closely resemble those of cast NAB reported in the literature. This similarity indicates that the crystallographic structure of the phases in cold-sprayed NAB are consistent with that of traditionally cast NAB. Such congruence in the XRD patterns suggests that the cold spray AM process successfully replicates the desirable microstructural characteristics of cast NAB, thereby ensuring comparable performance and reliability in practical applications.

Table 2 Tensile properties of NAB materials after HIP

	**Process**	**Average Yield Strength (MPa)**	**Average Ultimate Tensile Strength (MPa)**	**Average Elongation (%)**	**Average Hardness (Brinell, typ)**
**Nickel Aluminium Bronze**	Sintering, hipped and aging	289 ± 1.6	512 ± 2.7	22 ± 0.3	160 ± 1.8

Table 2 suggests the mechanical properties of hipped material exceed the ASTM specification for cast Nickel aluminium bronze (C95800). The enhancement in mechanical properties achieved through the addition of alloying elements and the subsequent heat treatment process highlights the potential of this approach for tailoring the alloy characteristics of cold-sprayed materials. The ability to approach the properties of conventionally cast alloys while leveraging cost-effective processing techniques, or exceed them where better techniques are available, presents promising opportunities for various applications.

Conclusions

The study shows the benefits of cold spray AM compared to traditional PM for making high-density materials, such as NAB parts. PM needs very high compaction pressures to achieve over 90% compact density. But cold spray AM can reach over 98% density with much less complexity in one off tooling and high pressure presses. This could save time and cost, making cold spray AM an interesting choice for industrial uses. The technique is also very flexible,

reducing the requirement for pre-alloyed powders and presenting significant scope for tailoring alloys.

The study indicates that the microstructure achieved through cold spray AM for both materials is comparable to that of cast materials after appropriate heat treatment. In the case of aluminium bronze, the dominant alpha phase, along with a minor presence of beta phase, contributes to an overall increase in material strength. Additionally, kappa phase precipitation in NAB enhances the tensile strength of the material. The study also shows that post-spray heat treatments, such as HIP, can further enhance the mechanical properties of cold-sprayed NAB samples, making them superior to traditionally cast NAB materials. This highlights the potential of cold spray AM to produce high-performance materials more efficiently. Thus, in general, cold spray AM offers significant advantages over PM, including improved process efficiency, material properties, and cost-effectiveness. It presents a promising method for producing complex and high-strength materials, warranting further research to optimize its parameters and expand its applications.

Statements and Declarations

Acknowledgments

The authors would like to acknowledge the support of the technical staff at Charles Darwin University.

Funding statement

This research was supported (partially or fully) by the Australian Government through the Department of Education's National Industry PhD Program (NIPhD 35017). The views expressed herein are those of the authors and are not necessarily those of the Australian Government or the Department of Education.

The work was partly funded by the generous support of Penn State ARL and SPEE3D.

There was no additional external funding received for this study." in your updated Funding Statement.

The funder provided support in the form of salaries for authors TT, SC and AD, but did not have any additional role in the study design, data collection and analysis, decision to publish, or preparation of the manuscript. The specific roles of these authors are articulated in the 'author contributions' section.

Authors' Contributions

S Camilleri carried out the design of experiments, analysis of data and writing of manuscript. T Tran conducted the experiments, analysis of data and editing the manuscript; A Duguid contributed to the design of experiments and analysis of data. K Krishnan contributed to the analysis of data, supervision and editing of manuscript.

Competing interests

S.C., T.T., and A.D. are employees of the SPEE3D. This does not alter our adherence to PLOS ONE policies on sharing data and materials.

Data Availability

All relevant data are within the manuscript and supporting information. We confirm that the submission contains all raw data required to replicate the results of the study.

Supporting information

S1 Data. Minimal underlying dataset of the study.

References

1. Richardson, I. *Guide to Nickel Aluminium Bronze for Engineers*. 2024 19/04/2024]; Available from: https://www.copper.org/applications/marine/nickel_al_bronze/pub-222-nickel-al-bronze-guide-engineers.pdf.
2. Schüssler, A. and H.E. Exner, *The corrosion of nickel-aluminium bronzes in seawater—I. Protective layer formation and the passivation mechanism.* Corrosion Science, 1993. **34**(11): p. 1793-1802. https://doi.org/10.1016/0010-938X(93)90017-B
3. Weston, G.M., *Survey of Nickel-Aluminium-Bronze Casting Alloys on Marine Applications, Australia Dept. of Defence Report,* . 1981, DSTO MRL: Melbourne, Victoria. https://apps.dtic.mil/sti/pdfs/ADA105263.pdf
4. Murray, T., et al., *Selective laser melting of nickel aluminium bronze.* Additive Manufacturing, 2020. **33**: p. 101122. https://doi.org/10.1016/j.addma.2020.101122
5. Morshed-Behbahani, K., et al., *Perspectives on the unusual electrochemical corrosion of Nickel Aluminum Bronze (NAB) alloy fabricated through laser-powder bed fusion additive manufacturing.* Corrosion Science, 2024. **228**: p. 111846. https://doi.org/10.1016/j.corsci.2024.111846
6. Fuller, M.D., et al., *Microstructural transformations and mechanical properties of cast NiAl bronze: Effects of fusion welding and friction stir processing.* Materials Science and Engineering: A, 2007. **463**(1): p. 128-137. https://doi.org/10.1016/j.msea.2006.07.157
7. Dharmendra, C., et al., *Microstructural evolution and mechanical behavior of nickel aluminum bronze Cu-9Al-4Fe-4Ni-1Mn fabricated through wire-arc additive manufacturing.* Additive Manufacturing, 2019. **30**. https://doi.org/10.1016/j.addma.2019.100872
8. Orzolek, S.M., J.K. Semple, and C.R. Fisher, *Influence of processing on the microstructure of nickel aluminum bronze (NAB).* Additive Manufacturing, 2022. **56**: p. 102859. https://doi.org/10.1016/j.addma.2022.102859
9. Ding, D., et al., *Fabricating Superior NiAl Bronze Components through Wire Arc Additive Manufacturing.* Materials, 2016. **9**(8): p. 652. https://doi.org/10.3390/ma9080652
10. Zhai, W., et al., *Additively manufactured (Fe, Ni)Al-reinforced nickel aluminum bronze with nearly-isotropic mechanical properties in build and transverse directions.* Materials Characterization, 2022. **184**: p. 111706. https://doi.org/10.1016/j.matchar.2021.111706
11. Öztürk, S., et al., *Improvement of microstructure, tribology and corrosion characteristics of nickel-aluminum bronze by P/M method.* Tribology International, 2020. **151**: p. 106519. https://doi.org/10.1016/j.triboint.2020.106519
12. Gera, D., et al., *Comparison of Cu–Al–Ni–Mn–Zr shape memory alloy prepared by selective laser melting and conventional powder metallurgy.* Transactions of Nonferrous Metals Society of China, 2020. **30**(12): p. 3322-3332. https://doi.org/10.1016/S1003-6326(20)65464-4
13. Chen, Y.T., et al., *A Review on Metallic Alloys Fabrication Using Elemental Powder Blends by Laser Powder Directed Energy Deposition Process.* Materials, 2020. 13(16). https://doi.org/10.3390/ma13163562
14. Prashar, G. and H. Vasudev, *A comprehensive review on sustainable cold spray additive manufacturing: State of the art, challenges and future challenges.* Journal of Cleaner Production, 2021. **310**: p. 127606. https://doi.org/10.1016/j.jclepro.2021.127606
15. Peng, Y.H., et al., *Effect of heat treatment on the gas-atomized nickel-aluminum bronze feedstock powders for cold spray.* Surface & Coatings Technology, 2024. **478**. https://doi.org/10.1016/j.surfcoat.2024.130458
16. Rokni, M.R., C.A. Widener, and V.R. Champagne, *Microstructural Evolution of 6061 Aluminum Gas-Atomized Powder and High-Pressure Cold-Sprayed Deposition.*

Journal of Thermal Spray Technology, 2014. **23**(3): p. 514-524. https://doi.org/10.1007/s11666-013-0049-y
17. Deng, Z., et al., *Microstructure and mechanical properties of Cu–12Al–xNi alloy prepared using powder metallurgy.* Materials Science and Engineering: A, 2019. **759**: p. 241-251. https://doi.org/10.1016/j.msea.2019.05.051
18. Ziel, R., A. Haus, and A. Tulke, *Quantification of the pore size distribution (porosity profiles) in microfiltration membranes by SEM, TEM and computer image analysis.* Journal of membrane science, 2008. **323**(2): p. 241-246. https://doi.org/10.1016/j.memsci.2008.05.057
19. Martin III, W.D., B.J. Putman, and N.B. Kaye, *Using image analysis to measure the porosity distribution of a porous pavement.* Construction and Building Materials, 2013. **48**: p. 210-217. https://doi.org/10.1016/j.conbuildmat.2013.06.093
20. Gohar, G.A., T. Manzoor, and A.N. Shah, *Investigation of thermal and mechanical properties of Cu-Al alloys with silver addition prepared by powder metallurgy.* Journal of Alloys and Compounds, 2018. **735**: p. 802-812. https://doi.org/10.1016/j.jallcom.2017.11.176
21. Kumar, N., et al., *Effect of powder metallurgy process and its parameters on the mechanical and electrical properties of copper-based materials: Literature review.* Powder Metallurgy and Metal Ceramics, 2020. **59**: p. 401-410. https://doi.org/10.1007/s11106-020-00174-1
22. Kumar, N., A. Bharti, and K.K. Saxena, *A re-investigation: Effect of powder metallurgy parameters on the physical and mechanical properties of aluminium matrix composites.* Materials Today: Proceedings, 2021. **44**: p. 2188-2193. https://doi.org/10.1016/j.matpr.2020.12.351
23. Dixit, M., and R. K. Srivastava, *Effect of compaction pressure on microstructure, density and hardness of Copper prepared by Powder Metallurgy route.* In IOP conference series: materials science and engineering, 2018. **377**: p. 012209. 10.1088/1757-899X/377/1/012209
24. German, R.M., *Powder metallurgy science.* (No Title), 1984. https://cir.nii.ac.jp/crid/1130000797654780032
25. Yusoff, M. and Z. Hussain, *Effect of compaction pressure on microstructure and properties of copper-based composite prepared by mechanical alloying and powder metallurgy.* Advanced Materials Research, 2013. 795: p. 343-346. https://doi.org/10.4028/www.scientific.net/AMR.795.343
26. Maev, R. and V. Leshchynsky, *Cold-spray coatings: recent trends and future perspectives.* Low-Pressure Cold Spray, 2018: p. 95-142. https://doi.org/10.1007/978-3-319-67183-3_4
27. Fan, N., et al., *A new strategy for strengthening additively manufactured cold spray deposits through in-process densification.* Additive Manufacturing, 2020. **36**: p. 101626. https://doi.org/10.1016/j.addma.2020.101626
28. Vo, P., et al., *Mechanical and microstructural characterization of cold-sprayed Ti-6Al-4V after heat treatment.* Journal of thermal spray technology, 2013. **22**: p. 954-964. https://doi.org/10.1007/s11666-013-9945-4
29. Li, W.-Y., et al., *Significant influences of metal reactivity and oxide films at particle surfaces on coating microstructure in cold spraying.* Applied Surface Science, 2007. **253**(7): p. 3557-3562. https://doi.org/10.1016/j.apsusc.2006.07.063
30. Li, Y.-J., X.-T. Luo, and C.-J. Li, *Dependency of deposition behavior, microstructure and properties of cold sprayed Cu on morphology and porosity of the powder.* Surface and Coatings Technology, 2017. **328**: p. 304-312. https://doi.org/10.1016/j.surfcoat.2017.08.070
31. Bill, W., et al. *Powder metallurgy and solid state processing of Armstrong Titanium and Titanium alloy powders*. in *ITA Annual Conference*. 2007.
32. MacDonald, D., et al., *Cold spraying of armstrong process titanium powder for additive manufacturing.* Journal of thermal spray technology, 2017. **26**: p. 598-609. https://doi.org/10.1007/s11666-016-0489-2

33. Ivasishin, O.M., et al., *Cost-effective blended elemental powder metallurgy of titanium alloys for transportation application.* Key Engineering Materials, 2000. 188: p. 55-62. https://doi.org/10.4028/www.scientific.net/KEM.188.55
34. Orzolek, S., J. Semple, and C. Fisher, *Nickel–Aluminum bronze (NAB) review: additive manufacturing and weldability.* Nav Surf Warf Cent Carderock Div, 2021. **2021**: p. 1.
35. Tran, T.T.T. and K. Krishnan, *Antimicrobial property, corrosion resistance and tarnish resistance of cold-sprayed additive manufactured copper-nickel alloy.* Progress in Additive Manufacturing, 2023: p. 1-12. https://doi.org/10.1007/s40964-023-00517-5
36. Kim, W., et al., *Optimization of strength and ductility of 2024 Al by equal channel angular pressing (ECAP) and post-ECAP aging.* Scripta Materialia, 2003. **49**(4): p. 333-338. https://doi.org/10.1016/S1359-6462(03)00260-4
37. Horita, Z., et al., *Achieving high strength and high ductility in precipitation-hardened alloys.* Advanced Materials, 2005. **17**(13): p. 1599-1602. 10.1002/adma.200500069
38. Zhao, Y.-H., et al., *Simultaneously increasing the ductility and strength of nanostructured alloys.* Advanced Materials, 2006. 18(17): p. 2280-2283. https://doi.org/10.1002/adma.200600310
39. Sudharshan Phani, P., et al., *Effect of process parameters and heat treatments on properties of cold sprayed copper coatings.* Journal of Thermal Spray Technology, 2007. **16**: p. 425-434. https://doi.org/10.1007/s11666-007-9048-1
40. Coddet, P., et al., *Effect of cold work, second phase precipitation and heat treatments on the mechanical properties of copper–silver alloys manufactured by cold spray.* Materials Science and Engineering: A, 2015. **637**: p. 40-47. https://doi.org/10.1016/j.msea.2015.04.008Get rights and content
41. Hutasoit, N., et al., *Effect of build orientation and post-build heat treatment on the mechanical properties of cold spray additively manufactured copper parts.* The International Journal of Advanced Manufacturing Technology, 2020. **110**: p. 2341-2357. https://doi.org/10.1007/s00170-020-06010-5
42. Li, W.-Y., et al., *Effect of heat treatment on the microstructure and microhardness of cold-sprayed tin bronze coating.* Applied surface science, 2007. **253**(14): p. 5967-5971. https://doi.org/10.1016/j.apsusc.2006.12.108

Click here to access/download;Figure;Fig1.tiff

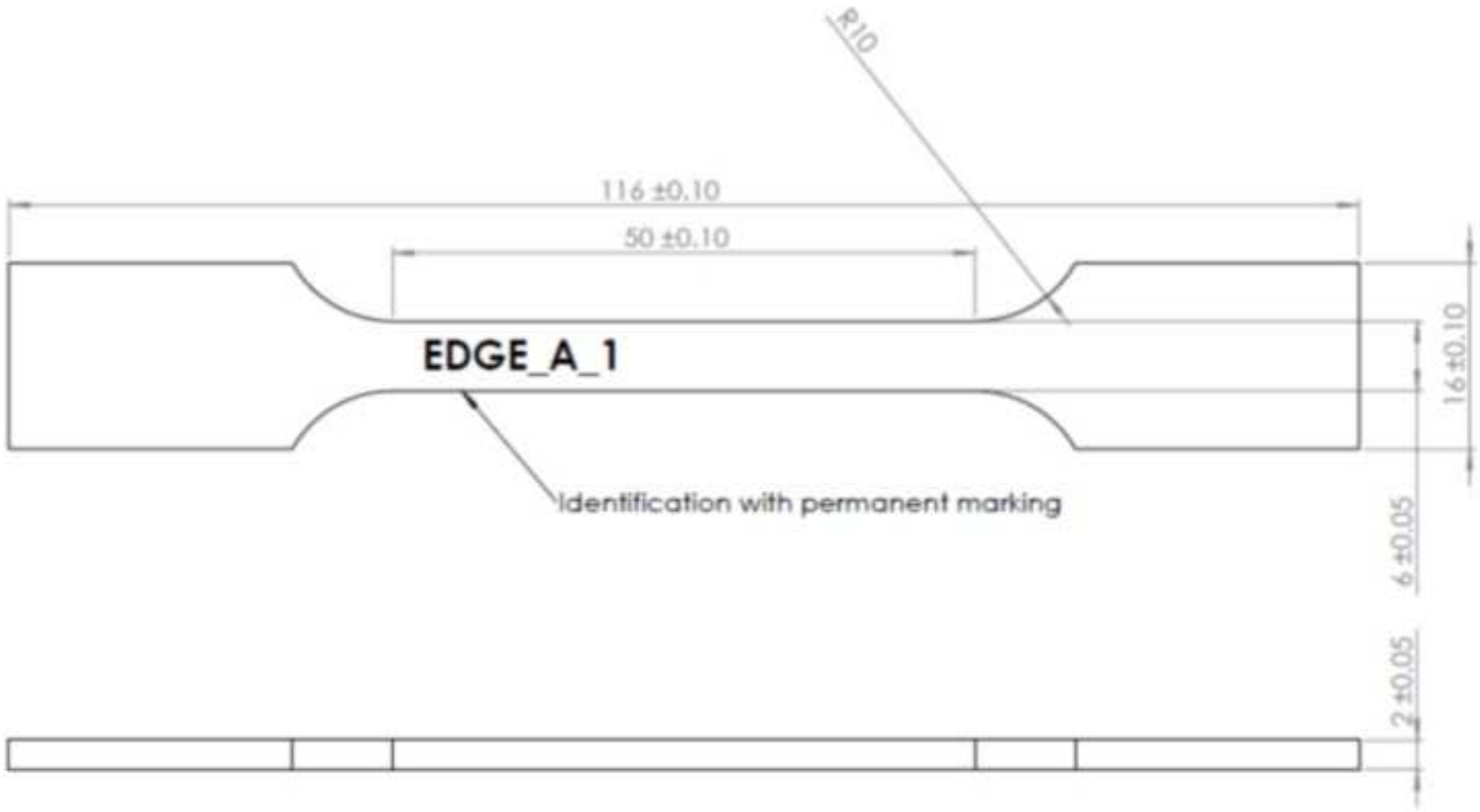

Click here to access/download;Figure;Fig2.tiff

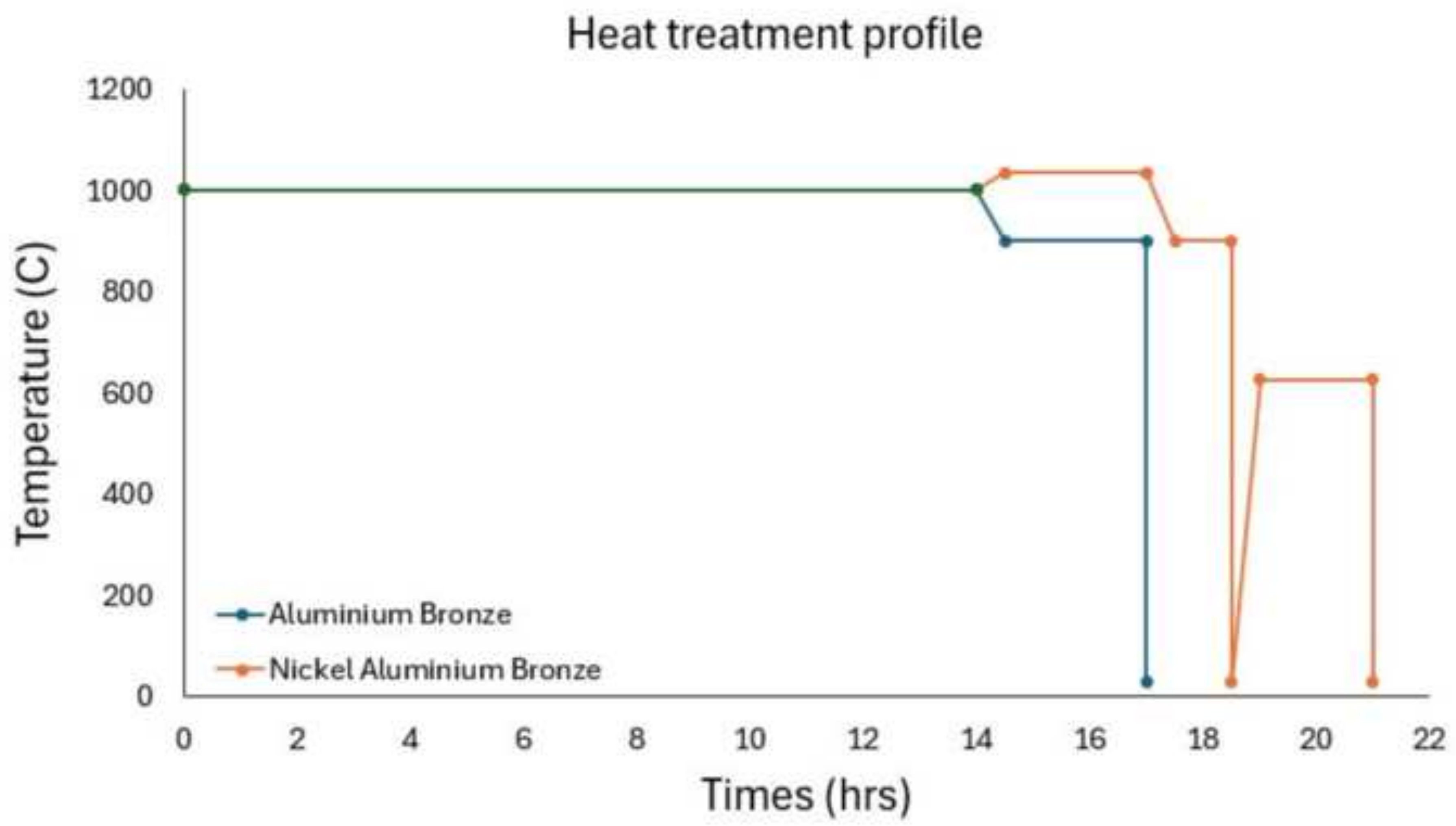

Fig3a

Click here to access/download;Figure;Fig3a.tiff

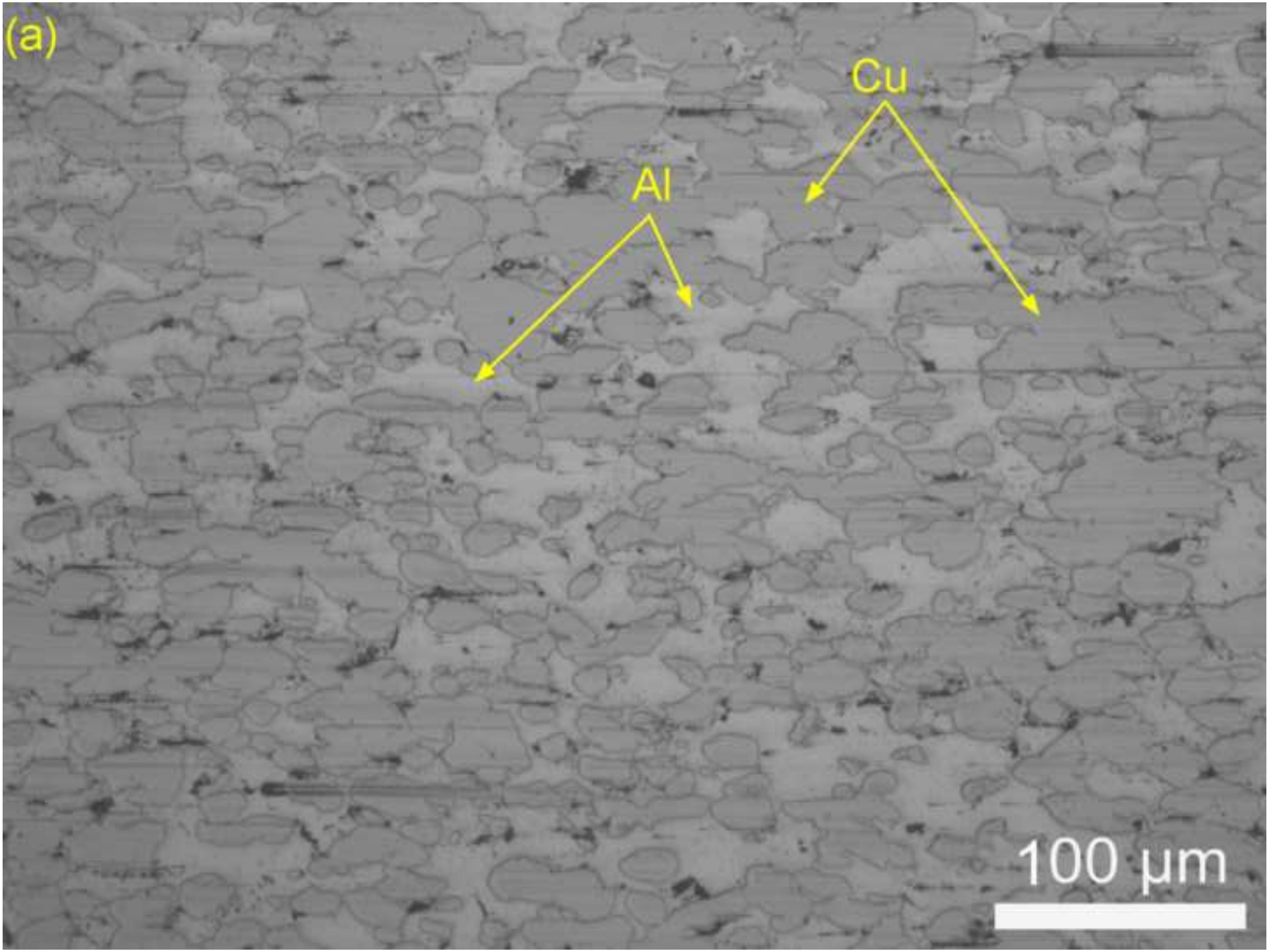

Fig3b

Click here to access/download;Figure;Fig3b.tiff

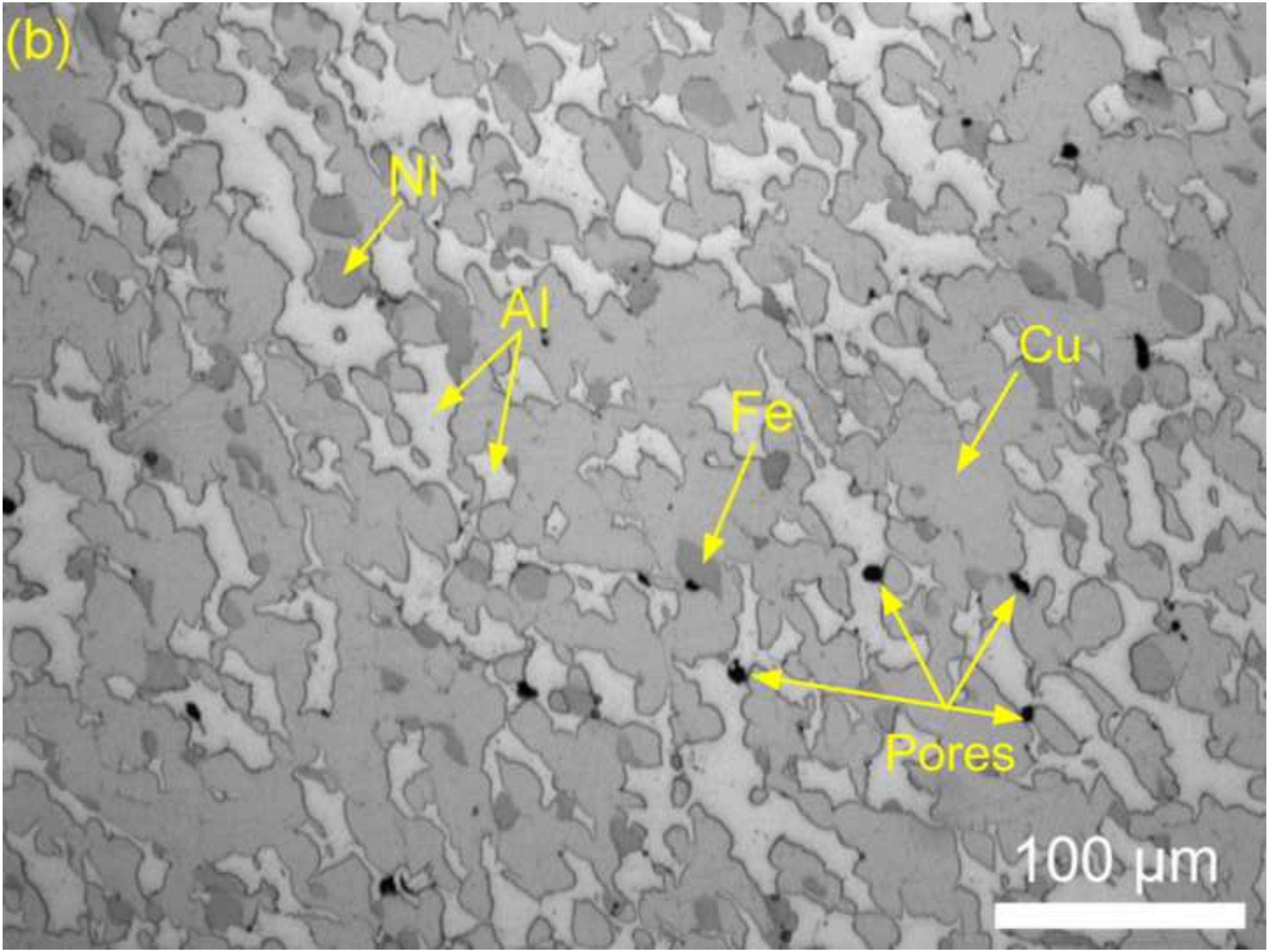

Fig4a

Click here to access/download;Figure;Fig4a.tiff

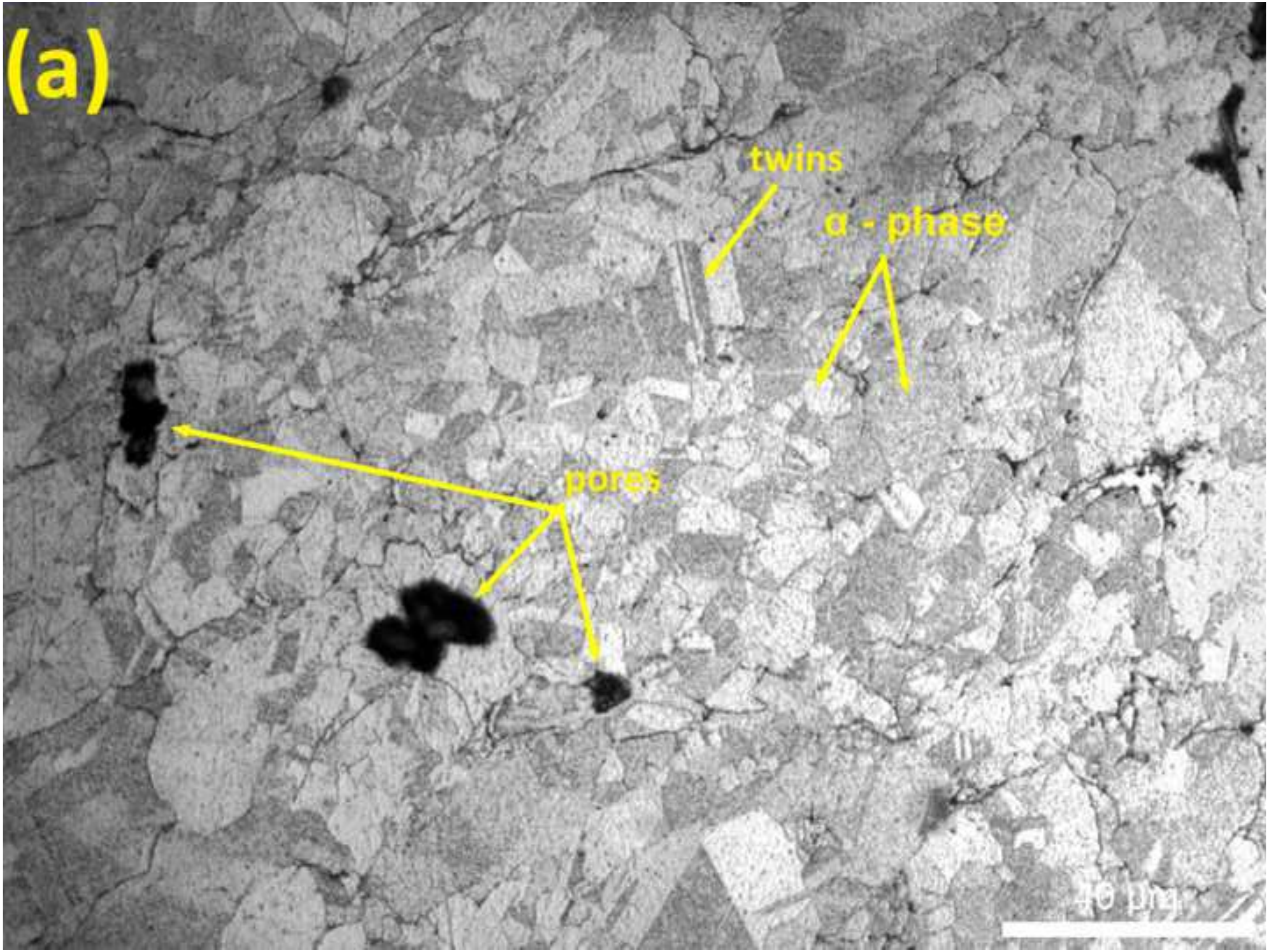

Fig4b

Click here to access/download;Figure;Fig4b.tiff

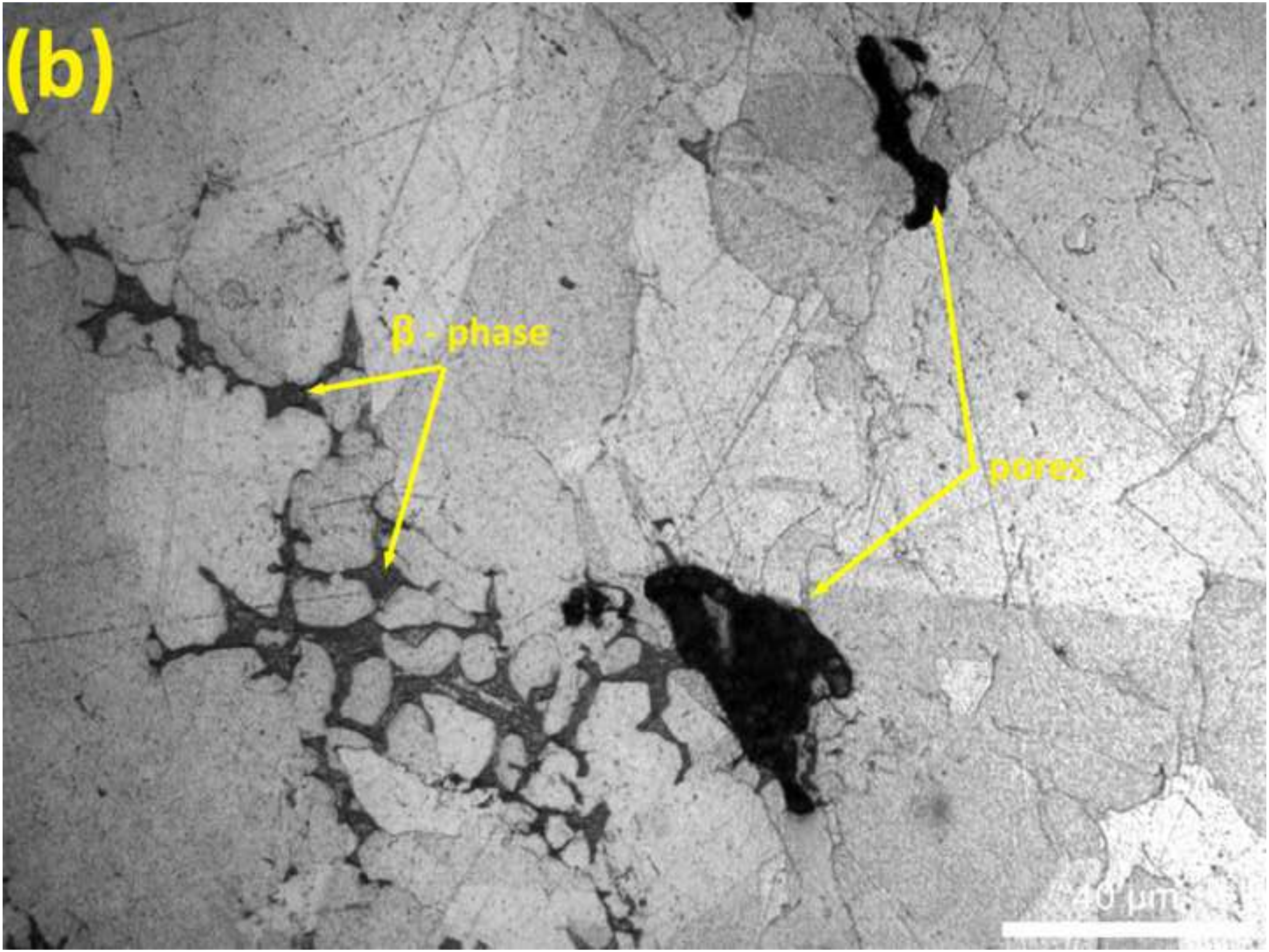

Fig4c

Click here to access/download;Figure;Fig4c.tiff

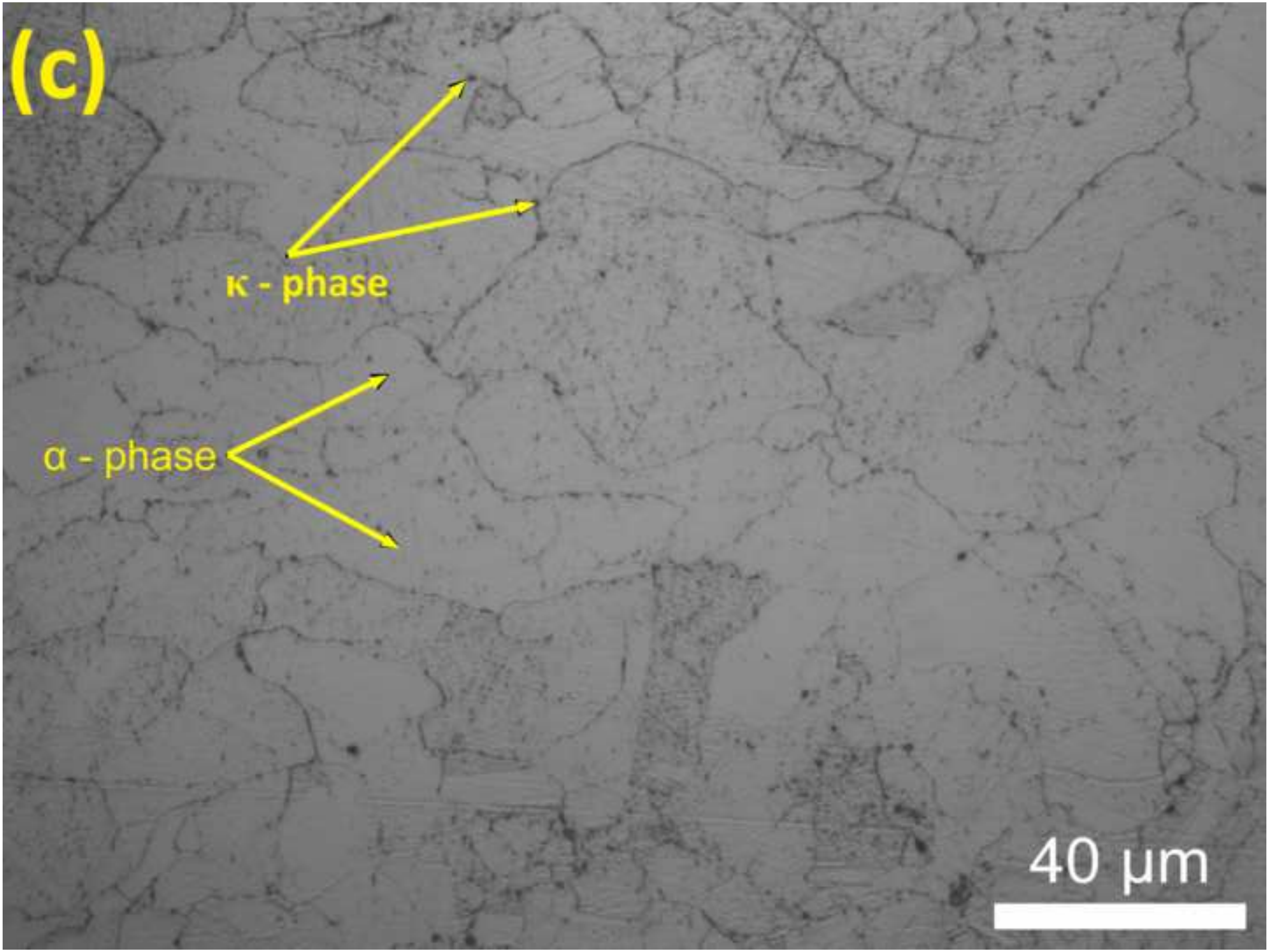

Fig5

Click here to access/download;Figure;Fig5.tiff

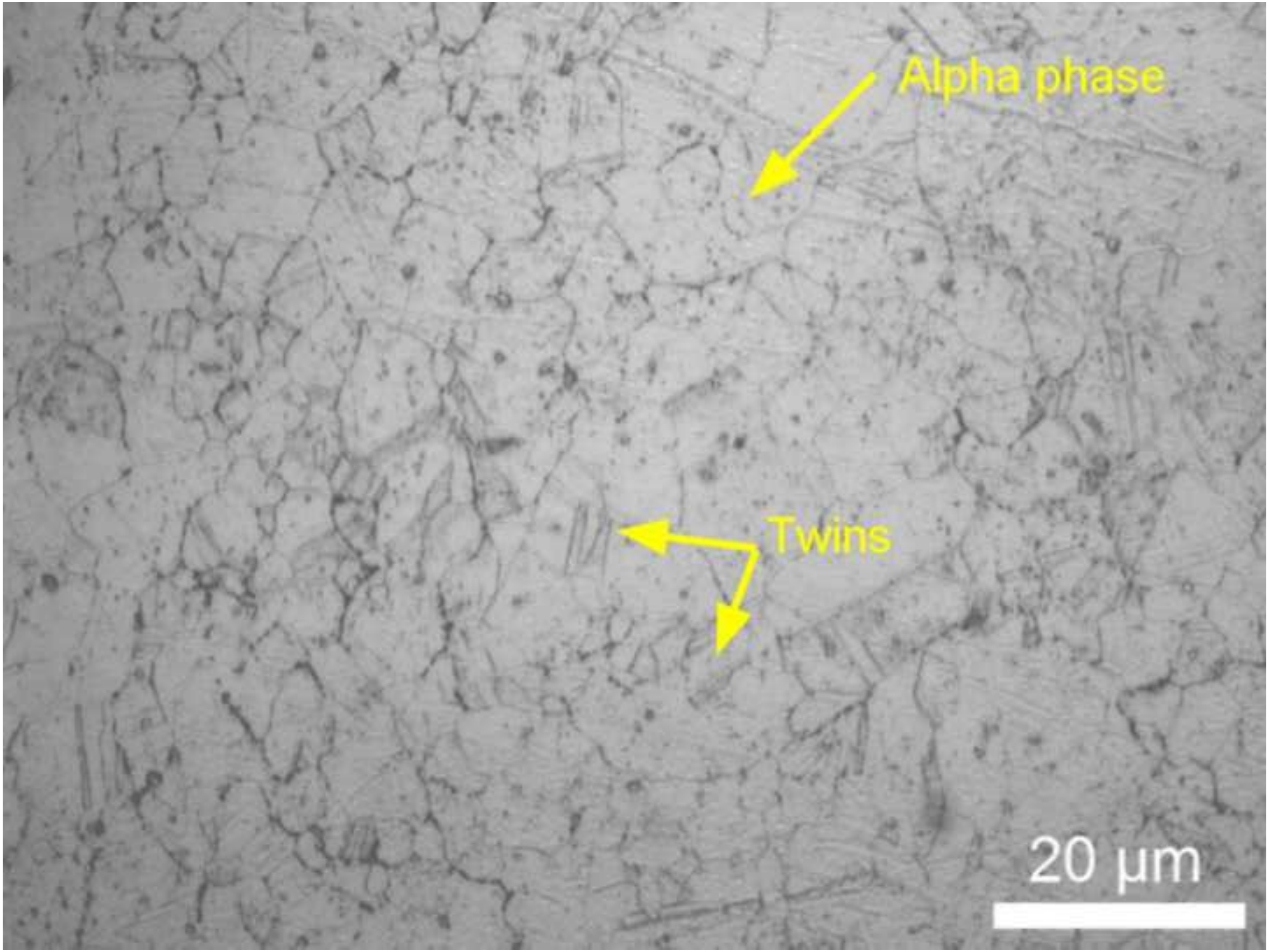

Fig6

Click here to access/download;Figure;Fig6.tiff

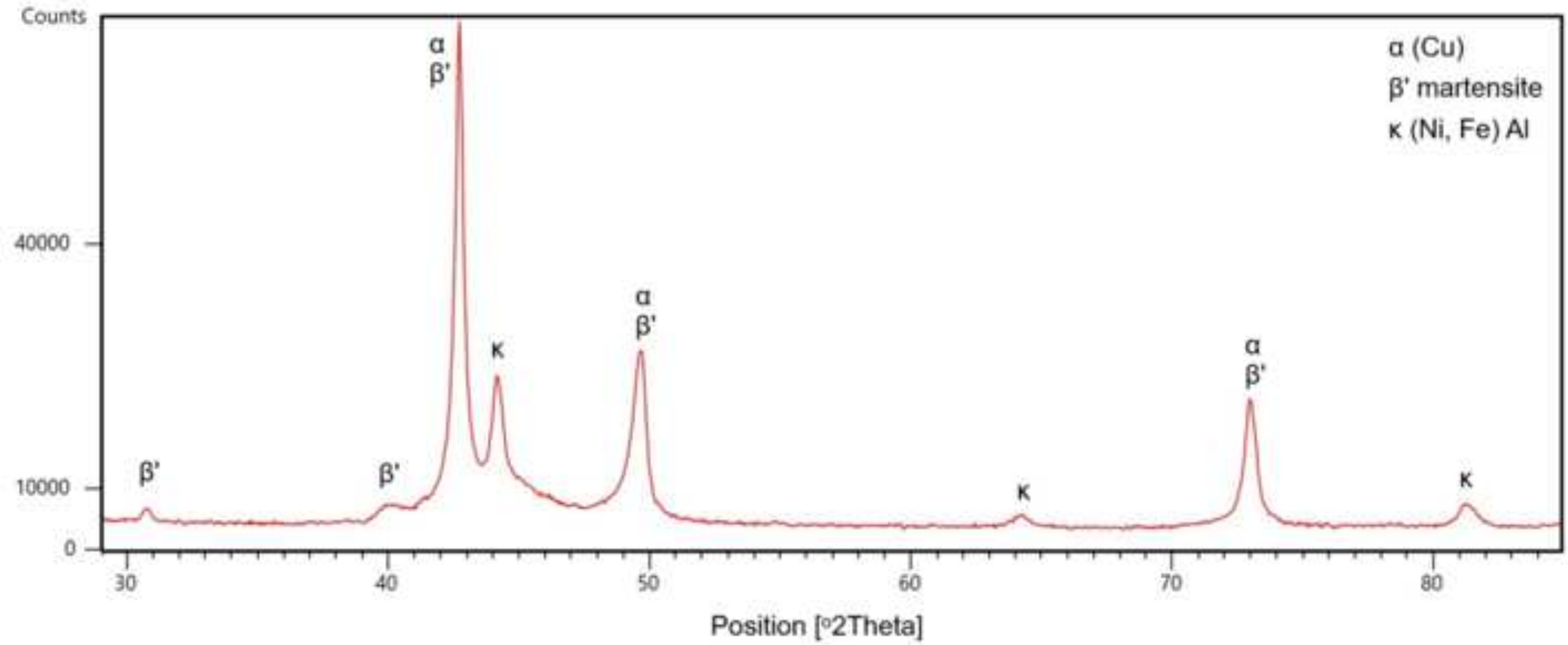

Click here to access/download
Supporting Information
S1 Data Minimal underlying dataset of the study.xlsx

Cold spray as a powder metallurgy process for production of nickel aluminium bronze

Formatted: Font: Bold, Italic

Steven Camilleri [1,2]¶, Tien T T Tran [1,2]¶, Andrew Duguid [2]¶, Krishnan Kannoorpatti [1*]

~~1.~~ [1] Advanced Manufacturing Alliance, Energy and Resources Institute, Charles Darwin University, Darwin, ~~NT 0909,~~ Australia

[2] SPEE3D, ~~PO Box U146,~~ Charles Darwin University, ~~NT 0815,~~ Australia

*Corresponding author

~~2.~~ E-mail: Krishnan.kannoorpatti@cdu.edu.au (KK)

Formatted: Normal, Indent: Left: 0.25", No bullets or numbering

Formatted: Normal, Indent: Left: 0.25", No bullets or numbering

Abstract

Nickel aluminium bronze (NAB) alloys are known for their excellent strength and corrosion resistance, making them suitable for maritime and industrial applications. NAB is producible by Powder Metallurgy (PM) but typically requires high compaction pressure. The objective of this study is to investigate the manufacturing of NAB using the cold spray additive manufacturing (AM) process and to compare its properties to those produced by traditional methods such as casting and PM. Cold spray is a solid-state coating technique that accelerates powdered metal and carrier gas to supersonic speeds, enabling bonding through plastic deformation. Binary aluminium bronze (AB) and NAB alloys were produced using powders by cold spraying powders into 3D printed parts, and heat treating the resulting parts. The AB alloy contained blended 9.9% aluminium alloy (Al6061) powder and copper powder, while the NAB alloy included 11% Al6061 powder, 5.8% nickel powder, 6.8% iron powder, and copper powder. Powders were mixed under controlled conditions and deposited using a LightSPEE3D printer and compressed air. Post-deposition heat treatments, such as homogenisation, aging, and/or hot isostatic pressing (HIP), were applied to enhance material properties. The results indicate that the cold spray process, combined with appropriate heat treatments, can produce NAB alloys with desirable microstructures containing fine κ phases and mechanical properties with above 280 MPa yield strength, 500 MPa tensile strength and 20% elongation which are comparable to those achieved by traditional cast methods, and superior to PM methods. This study demonstrates the viability of cold spray AM to enhance the production of complex high-strength alloys, offering significant advancements for maritime and industrial applications.

Formatted: Heading 1, Left, Space Before: 0 pt, After: 0 pt, Line spacing: single

Introduction

Metal manufacturing has evolved significantly over the centuries, transitioning from traditional methods like casting and forging to more sophisticated techniques such as sheet metal forming and powder metallurgy (PM). In recent decades, additive manufacturing (AM) of metal has emerged, becoming increasingly commercially viable and reliable, particularly over the last ten years.

Among these innovative methods, the c~~C~~old s~~S~~pray technique, also known as g~~G~~as d~~D~~ynamic c~~C~~old s~~S~~pray, stands out as a uniquely solid-state coating process that propels powdered metal and a carrier gas to supersonic speeds through a de Laval nozzle. Unlike traditional thermal spray techniques, which often lead to significant thermal degradation, oxidation, or phase transformations, c~~C~~old s~~S~~pray ensures that upon impact with a substrate, the particles undergo plastic deformation and bond to the surface or prior particles without significant heating. While AM is lauded for its ability to create objects with customisable geometries without the need for traditional tooling, it is less recognized for its ability to customise material properties. By mixing elemental metal powders rather than using prealloyed powders, various alloys can be created providing greater flexibility. This flexibility leads us to compare cold spray additive manufacturing with powder metallurgy. In this paper, the production of nickel aluminium bronze using cold spray method is compared with powder metallurgy process.

Nickel aluminium bronzes (NAB) are a popular set of alloys due to their excellent combination of properties. They have high strength and toughness, high wear, galling and cavitation resistances, are non-sparking, good corrosion and stress corrosion properties and biofouling resistance [1]. They are used extensively in aircraft landing gear bearings, marine propellers, pumps and valves, gears, and in non-sparking tools. They can be produced in cast, wrought or forged forms as well as by powder metallurgy. There are many different compositions of NAB alloy, but they generally contain up to 12% aluminium, 5% nickel, 7% iron, 3% manganese, and 2% silicon. The guide by Copper Development Association [1] is an excellent source of information on NAB. Aluminium is the primary element that strengthens the NAB alloys. Nickel and iron are added to improve corrosion and erosion resistance. Addition of iron also refines the structure and improves toughness. Manganese is added to improve fluidity of the alloys when casting.

When nickel and iron, about 5%, are added to copper-aluminium alloy, two phases α and κ are produced. When NAB is quenched from 900°C, both α phase, martensite β and κ phases can form. On tempering at 500-715°C, the martensite phase transforms to α (Cu-rich) and eutectoid phase (κ and γ_2) and secondary κ phases. The κ phase is found in 4 morphologies, locations and distributions. They are: K_I – rosette form and iron-rich, κ_{II} – spheroidised form at

the grain boundaries, κ_{III} – lath – shaped lamellar (Ni-rich) and κ_{IV} – fine precipitate inside grains and iron-rich. The precipitates being rich in certain elements tend to get selectively corroded [1] as these phases set up galvanic couples with the other phases.

The corrosion resistance of NAB has been attributed to the passive layers of oxides of aluminium and copper [2]. The passive layers also provide excellent resistance to flow induced corrosion due to the tenacity of the protective films.

Various kappa phases containing Ni and Fe are known to increase the mechanical properties of NAB. A decrease in elongation has been attributed to excess κ phases which form with an increase in aluminium content above nominal.

Iron more than 3 wt% reduces grain growth at high temperatures and produces a smaller grain size and solidification range. Addition of iron allows NAB to retain strength at high temperatures [3]. Nickel addition refines grains by retarding β phase formation. However, nickel content should not be lower than the iron content as this will lower corrosion resistance. A nominal content of 5 wt % Ni and 4 wt % Fe provides the optimal high strength along with corrosion resistance [3]. The properties of NAB are dependent on how well the phases are refined and distributed in the alloy. If the κ phases in which segregation of elements occurs can be reduced, then the properties will be more uniform [4].

This NAB alloy are produced mainly by casting processes. This can also be produced by PM processes. These can be fabricated using welding processes. Recently, the NAB alloys are being manufactured, by AM methods. Different manufacturing processes produce different microstructures and properties based on processing history.

Different manufacturing processes produce different microstructures and properties based on processing history. Selected Laser Melting (SLM) fabricated NAB alloys produced uniform martensitic structures with nano-sized Fe-rich particles (κ_{IV}) [5]. Annealing transformed the microstructure into α and lamellar/spheroidized Ni-rich particles. Martensite and κ_{IV} particles were not fully transformed.

Many of the Wire Arc Additive Manufacturing (WAAM) processes described in previous work significantly alter the microstructure of NAB producing grain growth and martensite in the heat affected zone [6-8]. The fusion zone exhibited Widmanstätten α and very fine martensite [9]. Martensite is harder and brittle and susceptible to corrosion and cavitation damage. After a Post-Weld Heat Treatment (PWHT) at 675°C for six hours refined the martensite to α and κ phases. The WAAM parts generally had better or equal mechanical properties to that of cast materials. κ_{I} was not precipitated due to the rapid cooling rates [7, 9].

Formatted: Font color: Auto

Formatted: Font color: Auto

Formatted: Font color: Red

In additive manufacturing using beam processes, the powders used are generally alloy powders [8, 10]. With the use of alloy powders, there is no flexibility to vary or compensate the composition of the alloy to get desired properties. Similarly, using ~~weld arc additive manufacturing (~~WAAM~~)~~, the wires used are standard wires and to make changes to the composition would require a whole new wire manufacturing run. Even powder metallurgy NAB products are manufactured using alloy powders [11, 12]. The lack of compositional flexibility can be addressed by using other processes that can produce the required NAB alloy from elemental powders. Chen et al [13] had provided a review of the use of elemental powders for laser powder directed energy deposition process. Some of the issues highlighted are that each elemental powder will reflect laser beam differently and their addition into the melt pool is inconsistent. This would require remelting of each layer a second time. In some cases, some of the elemental powders failed to melt and/or evaporated at different rates, leading to compositional error in the final alloy.

Cold spray AM is an emerging technique in AM that provides advantages for material deposition and alloy production. Cold spray AM operates as a solid-state process, using pressurised gas to accelerate metal powders at supersonic speeds onto a substrate. This allows the formation of a cohesive and dense layer without melting. Elemental powders can be blended and cold spayed and heat treated to produce complex alloys instead of using prealloyed powder. Cold spray AM is a suitable manufacturing method for powder with widely varying melting temperature, making it ideal for producing complex alloys with customised properties [14].

T~~However, t~~he use of elemental powders in ~~cold spray additive manufacturing (Cold Spray AM)~~cold spray AM provides opportunities for improving the production process and the properties of NAB alloys. This is because the process does not melt the material, so vastly differing melting temperatures of the elements do not present a concern. Additionally, the elemental powders are a lower cost than alloyed powders. Production of NAB alloys by ~~C~~cold s~~S~~pray AM has been attempted by Peng et al using NAB prealloyed powder [15]. It was found that the deposition efficiency was only 7.61% and it increased to 30.56% after annealing at the powder at 600°C for 10 hours. Heat treatment was done on the alloyed powder to modify the microstructure and to reduce the hardness. The deformation of hard, segregated alloy powder is less practical by cold spray unless a more expensive process relying on high pressure equipment or helium gas is used [16].

In this research, an attempt has been made to produce NAB which addresses some of the difficulties faced by other processes by using elemental powder, high pressure c~~C~~old s~~S~~pray AM method and using air as the propellant.

Materials and methodology

Materials feed stock

Aluminium bronze was selected for cold spray AM to take advantages of using elemental blended with widely different melting temperatures. A binary aluminium bronze (AB) was fabricated by using cold spray process with a mixed powder of elemental copper and aluminium alloy (Al6061). Separately a nickel aluminium bronze (NAB) alloy was fabricated by using cold spray process with a mixed powder comprised of elemental copper, aluminium alloy (Al6061), elemental nickel and elemental iron powders. The composition ratios for AB were 9.9 wt% aluminium alloy Al6061 and the remainder copper. For NAB, the ratios were 11 wt% aluminium alloy, 5.8 wt% nickel, 6.8 wt% iron, and the remainder copper. The powders used in this process were gas-atomised powder and were supplied by SPEE3D.

The powders were mixed by a V-blender powder mixing machine for 1.5 hours at room temperature. Argon gas was introduced during mixing to displace the air inside the chamber and reduce the formation of oxide during mixing. Powder moisture was controlled by adding silica gel desiccant into the powder mixer for further removing moisture.

2.2 Cold sSpray AM process

To form a cold spray compact, it is necessary to load the blended powder into the powder feeder of a cold spray AM apparatus, select a toolpath that will provide the right size and shape tensile specimen, set the cold spray process parameters, and then form the block. The cold spray AM apparatus chosen was a LightSPEE3D printer from the Australian company SPEE3D, equipped with their newer Phaser cold spray nozzle. The process gas was chosen to be compressed air in order to minimise process cost. The machine holds the cold-spray nozzle in a fixed position while a robot arm moves the workpiece over the particle-laden jet according to a deposition strategy determined by a proprietary 3D slicing algorithm. The carrier gas, air, is compressed to 30 bar, and heated to 540°C with 60 g/min. The standoff distance from the nozzle head to the build surface was fixed at 20 mm. Printed parts for tensile testing were rectangle blocks with dimensions of 20 mm x 32 mm x 106 mm in size. After forming the parts were subjected to different heat treatments and machined into groups of 5 tensile samples using EDM wire cutting (Fig. 1) according to ASTM E8.

Figure 1.1 Tensile sample design

Formatted: Font: 11 pt, Bold, Not Italic

Formatted: Font: 11 pt, Bold, Not Italic

Formatted: Left

Formatted: Font: 11 pt, Bold, Not Italic

Formatted: Font: 11 pt, Bold, Not Italic, Font color: Auto

2.3 Heat treatment process and testing

Press and sinter powder metallurgy relies on a wide range of sintering heat treatments to properly process a wide range of material systems. Advanced production PM furnaces are able to heat treat in hydrogen, or disassociated ammonia, or even inert atmospheres. In our case the sintering was carried out using an atmospheric furnace heat treatment (i.e., with minimum sintering cost) and in a separate experiment, using hot isostatic pressing using argon gas. This was done so a determination could readily be made if it was possible to obtain standard"normal" properties for the material system with an alternative processcasting.

Fig 2. Heat treatment profile for each material

The as-sprayed AB parts were homogenised at 1000°C for 14 hours and furnace cool to 900°C for 2.5 hours and quench in soluble oil. The as-sprayed NAB parts were homogenised at 1000°C for 14 hrs, increase temperature to 1035°C for 2.5 hours and furnace cool to 900°C for 1 hours and quench in soluble oil then followed by aging at 625°C for 2 hrs followed by quenching in soluble oil (Fig 2). With inclusion of HIP (Hot Isotactic Pressing) process, after the homogenisation step at 1000°C, the parts were then HIPed at 1035°C and 1000 bar for a 5-hour dwell, followed by a solution treatment at 1000°C to dissolve precipitates caused by the slow cooling process after HIPing. The parts were then aged at 625°C for 2hrs. The temperatures were chosen to produce sintering and homogeneous alloy based on previous literatures on PM on a similar alloy [17].

2.4 Metallography and X-ray Diffraction

The specimens were polished to 0.6µm finish and etched with Carpenter etchant ($FeCl_3$, $CuCl_2$, Hydrochloric acid Nitric acid and Ethanol). Microscopic observations were done using Nikon 600 optical microscope. Porosity of the as-sprayed samples were measured using imageJ analysis [18, 19]. The heat treated samples were studied using Xray diffraction (Malvern Panalytical) with copper X-ray target with the range from 25 to 90 degree at the rate of 4 degree per minute to study the phase components present.

Results and discussion

3.1 Relative density

Figure 23. Microstructure of as-sprayed alloy (a) Binary Aluminium Bronze (b) Nickel Aluminium Bronze

Formatted: Font: Bold

Formatted: Font: Bold

Formatted: Font: Bold

Formatted: Font: Bold, Not Italic

Formatted: Font: Bold

Formatted: Font: Bold, Not Italic

Formatted: Font: Bold

Formatted: Font: Bold, Not Italic

Fig. 23 shows the respective elements present in the right macro concentrationasconcentration as a metal matrix composite. The density porosity was measured to be about 982% (S1 Data) in the material which means that the density was about 98%. Visibly, aluminium distributed uniformly through the as-sprayed samples. In contrast, literature on the PM method shows the density reaches only 88% - 89.3% of a mixed powder of Cu-12Al-xNi which were compacted at 550 MPa and sintered at 1000°C [17]. Additionally, Cu-10Al-xAg also exhibit about 69% - 76% relative density with compact pressure at 200 MPa and sintered at 850°C [20]. This suggests the capability of cold spray AM to produce similar compacts density to PM but in higher density without the need for such high pressures.

It is known that traditional PM has ability to create high-density materials, typically in the range of 75% to 98% of the theoretical density, however this comes with significant limitations. The density and porosity of a product produced by the PM method is regulated by compaction pressure. The higher the compaction pressure, the greater the density, the lower the porosity, and the greater the final mechanical properties [21]. Mechanical, electrical, and other properties of a material are dependent on the density and porosity, which assumes, therefore, their indirect dependence on compaction pressure. Achieving densities above 90% typically requires very high compaction pressures (600-700 MPa) for aluminium – based material [22]. Additionally, the literature reports that iron and low-alloy steel powders require a compacted pressures at 600 MPa to 800 MPa to attain green densities of 85% to 90% of the pore-free solid density [23, 24]. Copper powders compacted at 600 MPa exhibit green densities of 88-93% of the theoretical density [23]. Mahani et al. examined the impact of compaction pressure on the hardness of Cu–W-C (graphite) alloy fabricated by powder metallurgy [25]. By increasing the compaction pressure, hardness increased due to a reduction in the porosity of the material.

In comparison, cold sprayed AM and coatings have also demonstrated high green densities which do not require high pressure compact process. Aluminium and aluminium alloy coatings have been shown to possess green densities in the range of 90-98% of the theoretical density [26]. Ningsong et. al. [27] indicated that the density of pure aluminium parts produced by cold spray can reach up to 99.93%. For titanium alloy Ti6Al4V, reported densities range from 70% to 99%: Vo et. al. reported 93% density with spraying in N_2 [28]; Wong et. al. reported 77.6% density with spraying in air [29]. Pure copper coatings have been reported to exhibit a density of at least 98% [30]. Cold spray AM of Amstrong process Titanium powder also exhibit as high density as PM process using the same powder pressed at above 700 MPa [31, 32]. Fully dense titanium parts were fabricated using the cold spray AM process. This was accomplished using low-pressure and low-temperature nitrogen as the process gas. The key to this result is

attributed to the unique coral-shaped powder morphology that accelerates well in the gas flow, allowing the easy densification and deposition through cold spray [32].

In powder metallurgy, it is common practice to use blended elemental powders or a mixture of a base metal powder and master alloy powders, rather than employing pre-alloyed powders of the desired composition [33]. This approach offers several advantages: it is more cost-effective than relying completely on pre-alloyed powders, it enables tailoring of properties such as particle size distribution and flowability, it facilitates better control of interdiffusion and homogeneity during sintering compared to pre-alloyed powders, and it provides flexibility in alloy design by allowing the production of compositions that may be difficult or impossible to obtain via melting and atomization of pre-alloyed powders. With blended powders, powder metallurgy has demonstrated high densities. Our study with cold spray AM also demonstrated high densities.

~~3.2~~ Microstructural characteristics

~~The heat treatment (HT)/sintering process is a crucial step in powder metallurgy involving mixed or blended powders, such as elemental powders or a combination of a base metal powder and master alloy powders. This thermal treatment facilitates interdiffusion and homogenization, enabling the formation of the desired alloy composition, phase constituents, and microstructure. The extent of diffusion bonding and grain growth depends on the sintering temperature and time. Higher temperatures improve the bonding between the particles and, hence, increases the strength of a material [19, 22] and densification [21]. Without proper heat treatment, the sintered components fabricated from mixed powders would exhibit incomplete alloying, high porosity, inhomogeneous microstructure, and inferior mechanical performance.~~

~~A similar phenomenon is observed in the cold spray process. The literature has demonstrated that heat treatment of cold-sprayed mixed powders, such as Cu-Ni, can help reduce the as-sprayed porosity and facilitate the formation of a homogenised alloy [31]. Literature also suggests that the strength of cold spray AM Aluminium alloy deposits can be increased by introducing small precipitates through performing heat treatment (HT) in age hardenable alloys, such as 2xxx, 6xxx, and 7xxx Al [32-34]. This is due to the fact that deformation before heat treatment is often used to promote non-uniform nucleation of precipitates in these alloys. Other CS copper and copper alloys are also reported to have better mechanical performance after HT [35-38]. Likewise, the current study shows cold spray AM aluminium bronze and nickel aluminium bronze can form homogenized alloys through a simple heat treatment process in a low-cost air atmosphere.~~

Formatted: Font: Bold

Formatted: Font: Bold

Formatted: Font: Bold

Formatted: Font: Bold

Formatted: Font: Bold

Formatted: Font: Bold

Formatted: Font: Bold

Formatted: Font: Bold

Field Code Changed

Formatted: Font: Bold

Formatted: Font: Bold

Field Code Changed

Formatted: Font: Bold

Figure 3 4. After Heat treatment, microstructure of (a-b) Binary Aluminium Bronze; (cb) Nickel Aluminium Bronze

Phases were identified according to literature review on microstructure of NAB [34]. The microstructural evolution and phase transformations observed in aluminium bronze and nickel aluminium bronze after heat treatment are illustrated in Fig. 34. Binary aluminium bronze shows the presence of both alpha phase as a major phase and beta phase (Figs 4 a and b). It is evident that the materials have undergone homogenization, resulting in the formation of well-defined alloy phases. The nickel aluminium bronze exhibits a predominantly single alpha phase microstructure with fine precipitates (Fig 4 c). The formation of these precipitates obtained by the aging process contributes to an increase in the material's tensile strength by means of precipitation hardening mechanisms. These microstructural observations highlight the efficacy of the heat treatment process in promoting interdiffusion, phase transformations, and the development of tailored microstructures, ultimately enhancing the mechanical properties of the respective aluminium bronze and nickel aluminium bronze alloys.

The heat treatment (HT)/sintering process is a crucial step in powder metallurgy involving mixed or blended powders, such as elemental powders or a combination of a base metal powder and master alloy powders. This thermal treatment facilitates interdiffusion and homogenization, enabling the formation of the desired alloy composition, phase constituents, and microstructure. The extent of diffusion bonding and grain growth depends on the sintering temperature and time. Higher temperatures improve the bonding between the particles and, hence, increases the strength of a material [22, 25] and densification [24]. Without proper heat treatment, the sintered components fabricated from mixed powders would exhibit incomplete alloying, high porosity, inhomogeneous microstructure, and inferior mechanical performance.

A similar phenomenon is observed in the cold spray process. The literature has demonstrated that heat treatment of cold-sprayed mixed powders, such as Cu-Ni, can help reduce the as-sprayed porosity and facilitate the formation of a homogenised alloy [35]. Literature also suggests that the strength of cold spray AM Aluminium alloy deposits can be increased by introducing small precipitates through performing heat treatment (HT) in age hardenable alloys, such as 2xxx, 6xxx, and 7xxx Al [36-38]. This is due to the fact that deformation before heat treatment is often used to promote non-uniform nucleation of precipitates in these alloys. Other CS copper and copper alloys are also reported to have better mechanical performance after HT [39-42]. Likewise, the current study shows cold spray AM aluminium bronze and nickel aluminium bronze can form homogenized alloys through a simple heat treatment process in a low cost air atmosphere.

Formatted: Font: Bold, Not Italic

Formatted: Font: Bold

Formatted: Font: Bold, Not Italic

Formatted: Font: Bold

Formatted: Font: Bold, Not Italic

Formatted: Font: Bold

Field Code Changed

Field Code Changed

~~3.3~~ Mechanical properties and further enhancing mechanical properties with the HIP process

To optimise the properties of the material, heat treatment processes were developed. The material properties post heat treatment is listed in Table 1. It should be noted that a constraint for total heat treatment time was introduced deliberately so that parts could be completely manufactured within a 24-hour cycle, including part printing, heat treatment etc. Consequently, the AB heat treatment was limited to 16.5 hours and the NAB heat treatment was limited to 18 hours. There was good evidence of the potential to improve the properties even further with longer heat treatments.

Table 1 Material properties with heat treatment only – no HIP

	Process	Average Yield Strength (MPa)	Average Ultimate Tensile Strength (MPa)	Average Elongation (%)	Average Hardness (Brinell)
**Binary Aluminium Bronze**	Sintering	160 ± 1.5~~160~~	300 ± 2.9~~300~~	8 ± 0.6~~8~~	87 ± 1.7~~87~~
**Nickel Aluminium Bronze**	Sintering and aging	257 ± 1.1~~257~~	404 ± 2.9~~404~~	10 ± 0.7~~10~~	150 ± 1.9~~150~~

The addition of alloying elements, such as Fe and Ni, plays a crucial role in enhancing the overall properties of the materials under consideration [17]. A higher concentration of these alloying elements contributes to an increase in tensile strength; however, it is accompanied by a reduction in elongation. Interestingly, despite the cost-effective approach adopted for heat treatment, the resulting material properties approach the properties specified by ASTM B505 cast nickel aluminium bronze standard.

Furthermore, other cold-sprayed compacts were subjected to atmospheric furnace heat treatments along with Hot Isostatic Pressing (HIP), leading to the microstructures shown in

Formatted: Heading 2, Left, Space Before: 0 pt, After: 0 pt, Line spacing: single

Formatted: Font: Bold

Formatted: Font: Bold, Not Italic

Formatted: Left

Formatted: Font: Bold, Not Italic

Formatted: Font: Bold, Not Italic

Fig .45. It is evident that the inclusion of a HIP process has resulted in reduced porosity and improved homogenization within the material.

Figure 4 5. Microstructure of HIPed Aluminium Bronze

Figure 5 6. XRD of HIPed Nickel Aluminium Bronze

The XRD analysis, shown in Fig .56, of the NAB produced by cCold sSpray AM revealed patterns that closely resemble those of cast NAB reported in the literature. This similarity indicates that the crystallographic structure of the phases in cold-sprayed NAB are consistent with that of traditionally cast NAB. Such congruence in the XRD patterns suggests that the cCold sSpray AM process successfully replicates the desirable microstructural characteristics of cast NAB, thereby ensuring comparable performance and reliability in practical applications.

Table 2 Tensile properties of NAB materials after HIP

	**Process**	**Average Yield Strength (MPa)**	**Average Ultimate Tensile Strength (MPa)**	**Average Elongation (%)**	**Average Hardness (Brinell, typ)**
**Nickel Aluminium Bronze**	Sintering, hipped and aging	289 ± 1.6289	512 ± 2.7512	22 ± 0.322	160 ± 1.8160

Table 2 suggests the mechanical properties of hipped material exceed the ASTM specification for cast Nickel aluminium bronze (C95800). The enhancement in mechanical properties achieved through the addition of alloying elements and the subsequent heat treatment process highlights the potential of this approach for tailoring the alloy characteristics of cold-sprayed materials. The ability to approach the properties of conventionally cast alloys while leveraging cost-effective processing techniques, or exceed them where better techniques are available, presents promising opportunities for various applications.

Conclusions

The study shows the benefits of cCold sSpray AM compared to traditional PM for making high-density materials, such as Nickel Aluminium BronzeNAB parts. PM needs very high compaction pressures to achieve over 90% compact density. But cCold sSpray AM can reach over 98% density with much less complexity in one off tooling and high pressure presses. This could save time and cost, making cCold sSpray AM an interesting choice for industrial uses.

Formatted: Font: Bold, Not Italic

Formatted: Font: Bold

Formatted: Font: Bold, Not Italic

Formatted: Font: Bold

Formatted: Font: Bold, Not Italic

Formatted: Font: Bold, Not Italic

The technique is also very flexible, reducing the requirement for pre-alloyed powders and presenting significant scope for tailoring alloys.

The study indicates that the microstructure achieved through cold spray AM for both materials is comparable to that of cast materials after appropriate heat treatment. In the case of aluminium bronze, the dominant alpha phase, along with a minor presence of beta phase, contributes to an overall increase in material strength. Additionally, kappa phase precipitation in NAB enhances the tensile strength of the material. The study also shows that post-spray heat treatments, such as HIP, can further enhance the mechanical properties of cold-sprayed NAB samples, making them superior to traditionally cast NAB materials. This highlights the potential of c~~C~~old s~~S~~pray AM to produce high-performance materials more efficiently. Thus, in general, c~~C~~old ~~S~~spray AM offers significant advantages over PM, including improved process efficiency, material properties, and cost-effectiveness. It presents a promising method for producing complex and high-strength materials, warranting further research to optimize its parameters and expand its applications.

Statements and Declarations

Formatted: Heading 1, Left, Space Before: 0 pt, After: 0 pt, Line spacing: single

Acknowledgments

The authors would like to acknowledge the support of the technical staff at Charles Darwin University.

Funding statement

Formatted: Heading 1, Left, Space Before: 0 pt, After: 0 pt, Line spacing: single

This research was supported (partially or fully) by the Australian Government through the Department of Education's National Industry PhD Program (NIPhD 35017). The views expressed herein are those of the authors and are not necessarily those of the Australian Government or the Department of Education.

The work was partly funded by the generous support of Penn State ARL and SPEE3D.

There was no additional external funding received for this study." in your updated Funding Statement.

The funder provided support in the form of salaries for authors TT, SC and AD, but did not have any additional role in the study design, data collection and analysis, decision to publish, or preparation of the manuscript. The specific roles of these authors are articulated in the 'author contributions' section.

Formatted: Font: Not Italic

Formatted: Not Highlight

Formatted: Not Highlight

Authors' Contributions

Formatted: Heading 1, Left, Space Before: 0 pt, After: 0 pt, Line spacing: single

S Camilleri carried out the design of experiments, analysis of data and writing of manuscript. T Tran conducted the experiments, analysis of data and editing the manuscript; A Duguid contributed to the design of experiments and analysis of data. K Krishnan contributed to the analysis of data, supervision and editing of manuscript.

Competing interests

Formatted: Heading 1, Left, Space Before: 0 pt, After: 0 pt, Line spacing: single

S.C., T.T., and A.D. are employees of the SPEE3D. This does not alter our adherence to PLOS ONE policies on sharing data and materials.

Formatted: Not Highlight

Formatted: Not Highlight

Data Availability

All relevant data are within the manuscript and supporting information. We confirm that the submission contains all raw data required to replicate the results of the study.

Supporting information

S1 Data. Minimal underlying dataset of the study.

Formatted: Heading 1, Left, Space Before: 0 pt, After: 0 pt, Line spacing: single

Formatted: Not Highlight

Formatted: Font: Bold

References

1. Richar~~ahr~~dson, I. *Guide to Nickel Aluminium Bronze for Engineers*. 2024 19/04/2024]; Available from: https://www.copper.org/applications/marine/nickel_al_bronze/pub-222-nickel-al-bronze-guide-engineers.pdf.
2. Schüssler, A. and H.E. Exner, *The corrosion of nickel-aluminium bronzes in seawater—I. Protective layer formation and the passivation mechanism.* Corrosion Science, 1993. **34**(11): p. 1793-1802. https://doi.org/10.1016/0010-938X(93)90017-B
3. Weston, G.M., *Survey of Nickel-Aluminium-Bronze Casting Alloys on Marine Applications, Australia Dept. of Defence Report,* . 1981, DSTO MRL: Melbourne, Victoria. https://apps.dtic.mil/sti/pdfs/ADA105263.pdf
4. Murray, T., et al., *Selective laser melting of nickel aluminium bronze.* Additive Manufacturing, 2020. **33**: p. 101122. https://doi.org/10.1016/j.addma.2020.101122
5. Morshed-Behbahani, K., et al., *Perspectives on the unusual electrochemical corrosion of Nickel Aluminum Bronze (NAB) alloy fabricated through laser-powder bed fusion additive manufacturing.* Corrosion Science, 2024. **228**: p. 111846. https://doi.org/10.1016/j.corsci.2024.111846
6. Fuller, M.D., et al., *Microstructural transformations and mechanical properties of cast NiAl bronze: Effects of fusion welding and friction stir processing.* Materials Science and Engineering: A, 2007. **463**(1): p. 128-137. https://doi.org/10.1016/j.msea.2006.07.157
7. Dharmendra, C., et al., *Microstructural evolution and mechanical behavior of nickel aluminum bronze Cu-9Al-4Fe-4Ni-1Mn fabricated through wire-arc additive manufacturing.* Additive Manufacturing, 2019. **30**. https://doi.org/10.1016/j.addma.2019.100872
8. Orzolek, S.M., J.K. Semple, and C.R. Fisher, *Influence of processing on the microstructure of nickel aluminum bronze (NAB).* Additive Manufacturing, 2022. **56**: p. 102859. https://doi.org/10.1016/j.addma.2022.102859
9. Ding, D., et al., *Fabricating Superior NiAl Bronze Components through Wire Arc Additive Manufacturing.* Materials, 2016. **9**(8): p. 652. https://doi.org/10.3390/ma9080652
10. Zhai, W., et al., *Additively manufactured (Fe, Ni)Al-reinforced nickel aluminum bronze with nearly-isotropic mechanical properties in build and transverse directions.* Materials Characterization, 2022. **184**: p. 111706. https://doi.org/10.1016/j.matchar.2021.111706
11. Öztürk, S., et al., *Improvement of microstructure, tribology and corrosion characteristics of nickel-aluminum bronze by P/M method.* Tribology International, 2020. **151**: p. 106519. https://doi.org/10.1016/j.triboint.2020.106519
12. Gera, D., et al., *Comparison of Cu–Al–Ni–Mn–Zr shape memory alloy prepared by selective laser melting and conventional powder metallurgy.* Transactions of Nonferrous Metals Society of China, 2020. **30**(12): p. 3322-3332. https://doi.org/10.1016/S1003-6326(20)65464-4
13. Chen, Y.T., et al., *A Review on Metallic Alloys Fabrication Using Elemental Powder Blends by Laser Powder Directed Energy Deposition Process.* Materials, 2020. 13(16). https://doi.org/10.3390/ma13163562
14. Prashar, G. and H. Vasudev, *A comprehensive review on sustainable cold spray additive manufacturing: State of the art, challenges and future challenges.* Journal of Cleaner Production, 2021. **310**: p. 127606. https://doi.org/10.1016/j.jclepro.2021.127606
15. Peng, Y.H., et al., *Effect of heat treatment on the gas-atomized nickel-aluminum bronze feedstock powders for cold spray.* Surface & Coatings Technology, 2024. **478**. https://doi.org/10.1016/j.surfcoat.2024.130458

Formatted: Font: Not Bold

Formatted: Font: Not Bold

16. Rokni, M.R., C.A. Widener, and V.R. Champagne, *Microstructural Evolution of 6061 Aluminum Gas-Atomized Powder and High-Pressure Cold-Sprayed Deposition.* Journal of Thermal Spray Technology, 2014. **23**(3): p. 514-524. https://doi.org/10.1007/s11666-013-0049-y
17. Deng, Z., et al., *Microstructure and mechanical properties of Cu–12Al–xNi alloy prepared using powder metallurgy.* Materials Science and Engineering: A, 2019. **759**: p. 241-251. https://doi.org/10.1016/j.msea.2019.05.051
18. Ziel, R., A. Haus, and A. Tulke, *Quantification of the pore size distribution (porosity profiles) in microfiltration membranes by SEM, TEM and computer image analysis.* Journal of membrane science, 2008. **323**(2): p. 241-246. https://doi.org/10.1016/j.memsci.2008.05.057
19. Martin III, W.D., B.J. Putman, and N.B. Kaye, *Using image analysis to measure the porosity distribution of a porous pavement.* Construction and Building Materials, 2013. **48**: p. 210-217. https://doi.org/10.1016/j.conbuildmat.2013.06.093
20. Gohar, G.A., T. Manzoor, and A.N. Shah, *Investigation of thermal and mechanical properties of Cu-Al alloys with silver addition prepared by powder metallurgy.* Journal of Alloys and Compounds, 2018. **735**: p. 802-812. https://doi.org/10.1016/j.jallcom.2017.11.176
21. Kumar, N., et al., *Effect of powder metallurgy process and its parameters on the mechanical and electrical properties of copper-based materials: Literature review.* Powder Metallurgy and Metal Ceramics, 2020. **59**: p. 401-410. https://doi.org/10.1007/s11106-020-00174-1
22. Kumar, N., A. Bharti, and K.K. Saxena, *A re-investigation: Effect of powder metallurgy parameters on the physical and mechanical properties of aluminium matrix composites.* Materials Today: Proceedings, 2021. **44**: p. 2188-2193. https://doi.org/10.1016/j.matpr.2020.12.351
23. Dixit, M., and R. K. Srivastava, *Effect of compaction pressure on microstructure, density and hardness of Copper prepared by Powder Metallurgy route.* In IOP conference series: materials science and engineering, 2018. **377**: p. 012209. 10.1088/1757-899X/377/1/012209
24. German, R.M., *Powder metallurgy science.* (No Title), 1984. https://cir.nii.ac.jp/crid/1130000797654780032
25. Yusoff, M. and Z. Hussain, *Effect of compaction pressure on microstructure and properties of copper-based composite prepared by mechanical alloying and powder metallurgy.* Advanced Materials Research, 2013. 795: p. 343-346. https://doi.org/10.4028/www.scientific.net/AMR.795.343
26. Maev, R. and V. Leshchynsky, *Cold-spray coatings: recent trends and future perspectives.* Low-Pressure Cold Spray, 2018: p. 95-142. https://doi.org/10.1007/978-3-319-67183-3_4
27. Fan, N., et al., *A new strategy for strengthening additively manufactured cold spray deposits through in-process densification.* Additive Manufacturing, 2020. **36**: p. 101626. https://doi.org/10.1016/j.addma.2020.101626
28. Vo, P., et al., *Mechanical and microstructural characterization of cold-sprayed Ti-6Al-4V after heat treatment.* Journal of thermal spray technology, 2013. **22**: p. 954-964. https://doi.org/10.1007/s11666-013-9945-4
29. Li, W.-Y., et al., *Significant influences of metal reactivity and oxide films at particle surfaces on coating microstructure in cold spraying.* Applied Surface Science, 2007. **253**(7): p. 3557-3562. https://doi.org/10.1016/j.apsusc.2006.07.063
30. Li, Y.-J., X.-T. Luo, and C.-J. Li, *Dependency of deposition behavior, microstructure and properties of cold sprayed Cu on morphology and porosity of the powder.* Surface and Coatings Technology, 2017. **328**: p. 304-312. https://doi.org/10.1016/j.surfcoat.2017.08.070
31. Bill, W., et al. *Powder metallurgy and solid state processing of Armstrong Titanium and Titanium alloy powders*. in *ITA Annual Conference*. 2007.

Formatted: Font: Not Bold

Formatted: Font: Not Bold

32. MacDonald, D., et al., *Cold spraying of armstrong process titanium powder for additive manufacturing.* Journal of thermal spray technology, 2017. **26**: p. 598-609. https://doi.org/10.1007/s11666-016-0489-2
33. Ivasishin, O.M., et al., *Cost-effective blended elemental powder metallurgy of titanium alloys for transportation application.* Key Engineering Materials, 2000. 188: p. 55-62. https://doi.org/10.4028/www.scientific.net/KEM.188.55
34. Orzolek, S., J. Semple, and C. Fisher, *Nickel–Aluminum bronze (NAB) review: additive manufacturing and weldability.* Nav Surf Warf Cent Carderock Div, 2021. **2021**: p. 1.
35. Tran, T.T.T. and K. Krishnan, *Antimicrobial property, corrosion resistance and tarnish resistance of cold-sprayed additive manufactured copper-nickel alloy.* Progress in Additive Manufacturing, 2023: p. 1-12. https://doi.org/10.1007/s40964-023-00517-5
36. Kim, W., et al., *Optimization of strength and ductility of 2024 Al by equal channel angular pressing (ECAP) and post-ECAP aging.* Scripta Materialia, 2003. **49**(4): p. 333-338. https://doi.org/10.1016/S1359-6462(03)00260-4
37. Horita, Z., et al., *Achieving high strength and high ductility in precipitation-hardened alloys.* Advanced Materials, 2005. **17**(13): p. 1599-1602. 10.1002/adma.200500069
38. Zhao, Y.-H., et al., *Simultaneously increasing the ductility and strength of nanostructured alloys.* Advanced Materials, 2006. 18(17): p. 2280-2283. https://doi.org/10.1002/adma.200600310
39. Sudharshan Phani, P., et al., *Effect of process parameters and heat treatments on properties of cold sprayed copper coatings.* Journal of Thermal Spray Technology, 2007. **16**: p. 425-434. https://doi.org/10.1007/s11666-007-9048-1
40. Coddet, P., et al., *Effect of cold work, second phase precipitation and heat treatments on the mechanical properties of copper–silver alloys manufactured by cold spray.* Materials Science and Engineering: A, 2015. **637**: p. 40-47. https://doi.org/10.1016/j.msea.2015.04.008Get rights and content
41. Hutasoit, N., et al., *Effect of build orientation and post-build heat treatment on the mechanical properties of cold spray additively manufactured copper parts.* The International Journal of Advanced Manufacturing Technology, 2020. **110**: p. 2341-2357. https://doi.org/10.1007/s00170-020-06010-5
42. Li, W.-Y., et al., *Effect of heat treatment on the microstructure and microhardness of cold-sprayed tin bronze coating.* Applied surface science, 2007. **253**(14): p. 5967-5971. https://doi.org/10.1016/j.apsusc.2006.12.108

Formatted: Font: Not Bold

Formatted: Font: Not Bold

Formatted: Font: Not Bold

Formatted: Font: Not Bold

Formatted: English (Australia)

Thank you for your constructive feedback on the manuscript titled " Cold spray as a powder metallurgy process for production of nickel aluminium bronze." Your insightful comments have significantly improved the manuscript, and I am grateful for your guidance.

Address to Editor's comments

Journal requirements:

When submitting your revision, we need you to address these additional requirements.

1. Please ensure that your manuscript meets PLOS ONE's style requirements, including those for file naming. The PLOS ONE style templates can be found at

https://journals.plos.org/plosone/s/file?id=wjVg/PLOSOne_formatting_sample_main_body.pdf and

https://journals.plos.org/plosone/s/file?id=ba62/PLOSOne_formatting_sample_title_authors_affiliations.pdf

- We have adjusted and corrected the manuscript to meet the journal requirements.

2. Thank you for stating in your Funding Statement:

"This research was supported (partially or fully) by the Australian Government through the Department of Education's National Industry PhD Program (NIPhD 35017). The views expressed herein are those of the authors and are not necessarily those of the Australian Government or the Department of Education.

The work was partly funded by the generous support of Penn State ARL and SPEE3D."

Please provide an amended statement that declares *all* the funding or sources of support (whether external or internal to your organization) received during this study, as detailed online in our guide for authors at http://journals.plos.org/plosone/s/submit-now. Please also include the statement "There was no additional external funding received for this study." in your updated Funding Statement.

Please include your amended Funding Statement within your cover letter. We will change the online submission form on your behalf.

- We have adjusted and corrected the manuscript to meet the journal requirements.

3. Thank you for stating the following financial disclosure:

“This research was supported (partially or fully) by the Australian Government through the Department of Education’s National Industry PhD Program (NIPhD 35017). The views expressed herein are those of the authors and are not necessarily those of the Australian Government or the Department of Education.

The work was partly funded by the generous support of Penn State ARL and SPEE3D.”

Please state what role the funders took in the study. If the funders had no role, please state: "The funders had no role in study design, data collection and analysis, decision to publish, or preparation of the manuscript."

If this statement is not correct you must amend it as needed.

Please include this amended Role of Funder statement in your cover letter; we will change the online submission form on your behalf.

- We have adjusted and corrected the manuscript to meet the journal requirements.

4. Thank you for stating the following in the Competing Interests

“I have read the journal's policy and the authors of this manuscript have the following competing interests:

Steven Camilleri, Tien Tran and Andrew Duguid are employees of SPEE3D. This has been declared in the authorship details.”

We note that one or more of the authors have an affiliation to the commercial funders of this research study : [SPEE3D].

a. Please provide an amended Funding Statement declaring this commercial affiliation, as well as a statement regarding the Role of Funders in your study. If the funding organization did not play a role in the study design, data collection and analysis, decision to publish, or preparation of the manuscript and only provided financial support in the form of authors' salaries and/or research materials, please review your statements relating to the author contributions, and ensure you have specifically and accurately indicated the role(s) that these authors had in your study. You can update author roles in the Author Contributions section of the online submission form.

Please also include the following statement within your amended Funding Statement.

“The funder provided support in the form of salaries for authors [insert relevant initials], but did not have any additional role in the study design, data collection and analysis, decision to publish, or preparation of the manuscript. The specific roles of these authors are articulated in the ‘author contributions’ section.”

If your commercial affiliation did play a role in your study, please state and explain this role within your updated Funding Statement.

b. Please also provide an updated Competing Interests Statement declaring this commercial affiliation along with any other relevant declarations relating to employment, consultancy, patents, products in development, or marketed products, etc.

Within your Competing Interests Statement, please confirm that this commercial affiliation does not alter your adherence to all PLOS ONE policies on sharing data and materials by including the following statement: "This does not alter our adherence to PLOS ONE policies on sharing data and materials.” (as detailed online in our guide for authors http://journals.plos.org/plosone/s/competing-interests). If this adherence statement is not accurate and there are restrictions on sharing of data and/or materials, please state these. Please note that we cannot proceed with consideration of your article until this information has been declared.

Please include both an updated Funding Statement and Competing Interests Statement in your cover letter. We will change the online submission form on your behalf.

- We have adjusted and corrected the manuscript to meet the journal requirements.

5. We note that your Data Availability Statement is currently as follows: [All relevant data are within the manuscript and its Supporting Information files.]

Please confirm at this time whether or not your submission contains all raw data required to replicate the results of your study. Authors must share the “minimal data set” for their submission. PLOS defines the minimal data set to consist of the data required to replicate all study findings reported in the article, as well as related metadata and methods (https://journals.plos.org/plosone/s/data-availability#loc-minimal-data-set-definition).

For example, authors should submit the following data:

- The values behind the means, standard deviations and other measures reported;

- The values used to build graphs;

- The points extracted from images for analysis.

- We confirm that the submission contains all raw data required to replicate the results of the study.

Thank you for your constructive feedback on the manuscript titled " Cold spray as a powder metallurgy process for production of nickel aluminium bronze." Your insightful comments have significantly improved the manuscript, and I am grateful for your guidance.

Address to reviewer's comments

Reviewer #1:

First of all, the paper is quite attractive since it touchs a very ineresting topic, in particular related to this complexes systems, NAB. It should be modify in order to point out the relevance of microstrcuture and the relation with the process per se and the impact on mechanical properties.

The microstrcutural analysis is poor.

- We have added more information on microstructure analysis into the manuscript.

Figures 3 and 4 needs to identify better the phases and microprepitates. So, have K phases this new cold spray AM new NAB?. Figure 3, how did you identify this "beta phase"?. It looks like rather defects.

- All phases identified in the etched microstructure as example from the literature.

Where is the Cu in Future 2?. what are this black spot of Figure 2 b?.

- We have added Cu arrow into the figures 2b to highlight the location of Cu. The black spots are all porosity. We also added this information into the figure.

Fig 3 and Figure 4 perhaps need more time for the etching in order to reveal better the microstructure, since it is the ley point of your research.

- We have etched the samples again and replaced the old images.

You mention "better properties " of your cold sprayed AM NABm, in comparison traditional methods. It should be interesting point out the relevance of phases, percentages, average of the grain size, distribution and so on.

- Thank you for your valuable suggestions. Due to limited facilities, we are unable to precisely measure phase quantities and grain size. The term "better properties" in the manuscript refers primarily to the material's properties. We have revised this section

in the manuscript to clarify and prevent any confusion.

The conclusions need to be reviewed in order to point out the real effect of microstructure.

- We have added more information in the conclusion section.

The references have some gramatical mistakes (ca ref 1).

- We have made correction.

Reviewer #2:

1. But cold spray also induces significant compressive force during deposition?
    - Yes. It is noted.
2. AM?
    - AM stands for Additive Manufacturing. We have made change in the manuscript.
3. “The results indicate that the cold spray process, combined with appropriate heat treatments, can produce NAB alloys with desirable microstructures and mechanical properties comparable to those achieved by traditional cast methods, and superior to PM methods.” – Examiner mentions, “This is a generic statement and does not provide any key information about the actual results of this study in both qualitative and quantitative values.”
    - We have made change in the manuscript as follows:

    “The results indicate that the cold spray process, combined with appropriate heat treatments, can produce NAB alloys with desirable microstructures containing fine κ phases and mechanical properties with above 280 MPa yield strength, 500 MPa tensile strength and 20% elongation which are comparable to those achieved by traditional cast methods, and superior to PM methods.”
4. Capitalised Cold Spray.
    - We have made change in the manuscript.
5. “Nickel aluminium bronzes (NAB) are a popular set of alloys due to their excellent combination of properties. They have high strength and toughness, high wear, galling and cavitation resistances, are non-sparking, good corrosion and stress corrosion properties and biofouling resistance [1]. They are used extensively in aircraft landing gear bearings, marine propellers, pumps and valves, gears, and in non-sparking tools. They can be produced in cast, wrought or forged forms as well as by powder metallurgy. There are many different compositions of NAB alloy, but they generally contain up to 12% aluminium, 5% nickel, 7% iron, 3% manganese, and 2% silicon. The guide by Copper Development Association [1] is an excellent source of information on NAB. Aluminium is the primary element that strengthens the NAB alloys. Nickel and iron are added to improve corrosion and erosion resistance. Addition of iron also refines the structure and improves toughness. Manganese is added to improve fluidity of the alloys when casting. When nickel and iron, about 5%, are added to copper-aluminium alloy, two phases α and κ are produced. When NAB is quenched from 900oC, both α phase, martensite β and κ phases can form. On tempering at 500-715oC, the martensite phase transforms to α (Cu-rich) and eutectoid phase (κ and γ2)

and secondary κ phases. The κ phase is found in 4 morphologies, locations and distributions. They are: KI – rosette form and iron-rich, κII – spheroidised form at". Examiner – "This information is primarily summarized from one source [1]. Please provide additional information from other published literature that can support these statements."

- We have added more information along with references in the manuscript.

6. "Various kappa phases containing Ni and Fe are known to increase the mechanical properties of NAB. A decrease in elongation has been attributed to excess κ phases which form with an increase in aluminium content above nominal." Examiner – "Please provide reference for this claim. Also mention the nominal aluminium content."
    - We have added more information along with references in the manuscript.
7. "WAAM" Examiner – "WAAM is mentioned in the previous paragraph as wire arc additive manufacturing."
    - We have made change in the manuscript.

8. "The first several paragraphs of the Introduction section was heavily focussed on microstructural phases exhibited in NAB alloys when subjected to different processing conditions. However, the later part of this section discussed more on additive manufacturing of NAB and the issues when printing using pre-alloyed powders. These two sections are quite distinct and there is no correlation drawn between microstructural variations in cast/forged/pht NAB parts with those of AMed NAB, as well as the mechanical property comparisions, which are the key results studied in this research work. Moreover, the Introduction lacks a pragraph on the research gaps and the importance/novelty of this study.
   In my opinion, this section has to be considerably improved to better present the state-of-the-art literature on CSAM, its application to not only NAB but other related Cu-alloys."
    - We have made changes in the manuscript.
9. Why was Aluminium bronze studied in this work? The rationale behind this alloy addition should be discussed in the Introduction section.
    - Aluminium bronze was selected to take advantages of using elemental blended with widely different melting temperatures.
10. What are the chemical compositions of the powders, their morphology, and their PSD? Was this the AL6061 alloy? What was the chemical composition of this powder? Is it 9.9% by weight or volume?

- Thank you for your insightful questions. We have revised the manuscript to include additional details about the powder, including its chemical composition. However, please note that the powder particle size distribution (PSD) is proprietary information, so we are unable to disclose it in the manuscript.

11. How long was the mixing carried out? Was it at room temperature? More experimental details are to be provided.
    - We have made changes in the manuscript.
12. What was the powder feed rate?
    - It is 60 g/min. We have made changes in the manuscript.
13. Were the same process parameters used to print both aluminium bronze and nickel aluminium bronze coupons?
    - Yes. The two materials were deposited with the same parameters.
14. Does normal properties in this instance refers to cast or wrought mechanical properties?
    - It refers to cast mechanical properties. We have changed the word normal to cast mechanical properties.
15. Why were these heat treatment temperatures selected? Can this heat treatment protocol be shown in the temperature schedule graph with respective heating and cooling rates? Also, was this HT followed for both AB and NAB?
    - We have added the HT for the AB and a graph in the manuscript. Additionally, we have added an explanation for the temperature chosen for heat treatment.
16. Please present a table to show how many conditions were studied and mechanically tested in this work?
    - Tables 1 and 2 provided the details.
17. For the XRD analysis, what was the x-ray source used? Also, the angular data capture rate?
    - We have made changes in the manuscript as below:

    The heat treated samples were studied using Xray diffraction (Malvern Panalytical) with copper X-ray target with the range from 25 to 90 degree at the rate of 4 degree per minute to study the phase components present.
18. There is no discussion of actual observations drawn from the micrographs shown in Figure 2.
    - We have made changes in the manuscript.
19. How was density measured? This is not discussed in the Methodology section.
    - The density was measured by 100% - %porosity. We have added this in the methodology section.

20. Does this statement refer to similar composition or similar properties?
    - The statement refers to similar density to PM. We have made changes to the statement to make it clearer.
21. This section is basically a discussion of published literature to compare relative densities achieved with that of PM process for various materials compared to that of cold spray process, which is relevant for an Introduction section. In this section, please discuss as to why the relative density of your NAB coupons in the as-sprayed condition is significantly higher than that reported in literature for other Cu-based cold sprayed parts. Is it owing to the nozzle design or process parameters or powder characteristics. A theory should be proposed and validated with subsequent experimentation. A recent study showed that the porosity percentage increases in heat treated aluminium bronze parts when compared with as-sprayed condition. https://doi.org/10.1016/j.nxmate.2024.100312
    - It is still unclear as to why we got high density for the materials. It could be attributed to the nozzle design, process parameters and powder characteristics. We have not use any other machine so we are unable to comment on comparing the density with others.
22. Why? Is it only for AB or NAB parts? More discussion of results is required in this section.
    - This statement refers to both materials.
23. The first two paragraphs of this section should be discussed after presenting the results of this study. So, Fig. 3 should be presented and the key observations discussed, followed by literature based discussions.
    - We have made changes to the manuscript.
24. How were the phases identified and correlated in the microstructure? Is there any EDS carried out to validate the statements made here.
    - The phases were identified by comparing with phases from literature.
25. Since 5 coupons were tensile tested at each condition, the standard deviation should be mentioned for all mean values shown in Table 1.
    - We have added this information.
26. Why was only sintering carried out for AB? Why not aging treatment?
    - There is no aging behaviour in AB system with 9.9%Al, thus no aging treatment was needed.

27. These properties should also be presented in Table 1 along with the mechanical properties of similar parts produced by CSAM for better comparision. Table 2 should be merged with Table 1 for better comparision.
    - These properties in different section, thus it is unsuitable to move the table to the previous section.
28. The Abstract mentions that the mechanical properties of cold sprayed NAB parts outperformed those of cast and PM parts. Where is this information presented in the Results and Discussion sections?
    - The statement was in section 3.3 as follows:

    “Table 2 suggests the mechanical properties of hipped material exceed the ASTM specification for cast Nickel aluminium bronze (C95800).”
