# Supplementary material for: Cold spray as a powder metallurgy process for production of nickel aluminium bronze
Source: PLoS One. 2025 Mar 4;20(3):e0319333. doi: 10.1371/journal.pone.0319333 (PMC11878897; doi:10.1371/journal.pone.0319333)
Supplement: Attachment — Submitted filename: Response to Editor.docx [file pone.0319333.s005.docx]

**Thank you for your constructive feedback on the manuscript titled "** **Cold spray as a powder metallurgy process for production of nickel aluminium bronze." Your insightful comments have significantly improved the manuscript, and I am grateful for your guidance.**

**Address to Editor’s comments**

Journal requirements:

When submitting your revision, we need you to address these additional requirements.

1. Please ensure that your manuscript meets PLOS ONE's style requirements, including those for file naming. The PLOS ONE style templates can be found at

<https://journals.plos.org/plosone/s/file?id=wjVg/PLOSOne_formatting_sample_main_body.pdf> and

<https://journals.plos.org/plosone/s/file?id=ba62/PLOSOne_formatting_sample_title_authors_affiliations.pdf>

- We have adjusted and corrected the manuscript to meet the journal requirements.

2. Thank you for stating in your Funding Statement:

“This research was supported (partially or fully) by the Australian Government through the Department of Education’s National Industry PhD Program (NIPhD 35017). The views expressed herein are those of the authors and are not necessarily those of the Australian Government or the Department of Education.

The work was partly funded by the generous support of Penn State ARL and SPEE3D.”

Please provide an amended statement that declares *all* the funding or sources of support (whether external or internal to your organization) received during this study, as detailed online in our guide for authors at <http://journals.plos.org/plosone/s/submit-now>.  Please also include the statement “There was no additional external funding received for this study.” in your updated Funding Statement.

Please include your amended Funding Statement within your cover letter. We will change the online submission form on your behalf.

- We have adjusted and corrected the manuscript to meet the journal requirements.

3. Thank you for stating the following financial disclosure:

“This research was supported (partially or fully) by the Australian Government through the Department of Education’s National Industry PhD Program (NIPhD 35017). The views expressed herein are those of the authors and are not necessarily those of the Australian Government or the Department of Education.

The work was partly funded by the generous support of Penn State ARL and SPEE3D.”

Please state what role the funders took in the study.  If the funders had no role, please state: "The funders had no role in study design, data collection and analysis, decision to publish, or preparation of the manuscript."

If this statement is not correct you must amend it as needed.

Please include this amended Role of Funder statement in your cover letter; we will change the online submission form on your behalf.

- We have adjusted and corrected the manuscript to meet the journal requirements.

4. Thank you for stating the following in the Competing Interests

“I have read the journal's policy and the authors of this manuscript have the following competing interests:

Steven Camilleri, Tien Tran and Andrew Duguid are employees of SPEE3D. This has been declared in the authorship details.”

We note that one or more of the authors have an affiliation to the commercial funders of this research study : [SPEE3D].

a. Please provide an amended Funding Statement declaring this commercial affiliation, as well as a statement regarding the Role of Funders in your study. If the funding organization did not play a role in the study design, data collection and analysis, decision to publish, or preparation of the manuscript and only provided financial support in the form of authors' salaries and/or research materials, please review your statements relating to the author contributions, and ensure you have specifically and accurately indicated the role(s) that these authors had in your study. You can update author roles in the Author Contributions section of the online submission form.

Please also include the following statement within your amended Funding Statement.

“The funder provided support in the form of salaries for authors [insert relevant initials], but did not have any additional role in the study design, data collection and analysis, decision to publish, or preparation of the manuscript. The specific roles of these authors are articulated in the ‘author contributions’ section.”

If your commercial affiliation did play a role in your study, please state and explain this role within your updated Funding Statement.

b. Please also provide an updated Competing Interests Statement declaring this commercial affiliation along with any other relevant declarations relating to employment, consultancy, patents, products in development, or marketed products, etc.

Within your Competing Interests Statement, please confirm that this commercial affiliation does not alter your adherence to all PLOS ONE policies on sharing data and materials by including the following statement: "This does not alter our adherence to  PLOS ONE policies on sharing data and materials.” (as detailed online in our guide for authors <http://journals.plos.org/plosone/s/competing-interests>). If this adherence statement is not accurate and  there are restrictions on sharing of data and/or materials, please state these. Please note that we cannot proceed with consideration of your article until this information has been declared.

Please include both an updated Funding Statement and Competing Interests Statement in your cover letter. We will change the online submission form on your behalf.

- We have adjusted and corrected the manuscript to meet the journal requirements.

5. We note that your Data Availability Statement is currently as follows: [All relevant data are within the manuscript and its Supporting Information files.]

Please confirm at this time whether or not your submission contains all raw data required to replicate the results of your study. Authors must share the “minimal data set” for their submission. PLOS defines the minimal data set to consist of the data required to replicate all study findings reported in the article, as well as related metadata and methods (https://journals.plos.org/plosone/s/data-availability#loc-minimal-data-set-definition).

For example, authors should submit the following data:

- The values behind the means, standard deviations and other measures reported;

- The values used to build graphs;

- The points extracted from images for analysis.

- We confirm that the submission contains all raw data required to replicate the results of the study.
