# Supplementary material for: Cold spray as a powder metallurgy process for production of nickel aluminium bronze
Source: PLoS One. 2025 Mar 4;20(3):e0319333. doi: 10.1371/journal.pone.0319333 (PMC11878897; doi:10.1371/journal.pone.0319333)
Supplement: Attachment — Submitted filename: Response to reviewers.docx [file pone.0319333.s006.docx]

**Thank you for your constructive feedback on the manuscript titled "** **Cold spray as a powder metallurgy process for production of nickel aluminium bronze." Your insightful comments have significantly improved the manuscript, and I am grateful for your guidance.**

**Address to reviewer’s comments**

**Reviewer #1:**

It should be mandatory specify in conclusion the heat treatments that you argued in the way "The study indicates that the microstructure achieved through cold spray AM for both materials is comparable to that of cast materials after appropriate heat treatment. ". Which heat treatment?. in which way is altering the microstructure?. does affected to beta/alphase percentages phases? K distribution ? percentajes ? shapes ? and so on.
**Answer:** Thank you for your insightful comments. We agree that the heat treatment details need to be explicitly stated in the conclusion. We will modify the conclusion to specify the heat treatments applied and their impact on microstructure, particularly regarding the α/β phase distribution and κ phase morphology.

Are you sure that you can identify the chemical elements defined in Figure 3?

**Answer:** Yes. We acknowledge the need to clarify the methodology used for elemental identification in Figure 3. The elements were identified through EDS, which was conducted on selected areas of the sample. To ensure accuracy, we will explicitly mention this method in the manuscript.

You conclude that "This highlights the potential of cCold sSpray AM to produce high-performance materials more efficiently. ". Do you consider that defects ( pores, shrinkages, etc) can penalize the performance of this technique?

**Answer:** Yes, defects such as porosity and incomplete bonding can influence the mechanical performance of cold spray AM materials. However, our study demonstrates that post-processing techniques, including heat treatment and hot isostatic pressing (HIP), significantly reduce porosity and improve mechanical properties. We will add a discussion on how these defects can be mitigated.

**Reviewer #2:**

1. Another useful publication to consider for this section: <https://doi.org/10.1016/j.nxmate.2024.100312>

**Answer:** We have added this into the manuscript. Thank you for the suggestion.

2. The abstract mentions that the study aims to compare NAB manufactured by cold spray AM to those produced by traditional methods (casting and PM), but the discussion lacks critical comparison.

**Answer:** Thank you for pointing this out. We have discussed in above sections, however we agree that is not enough. A further detailed comparison of the mechanical properties between cold spray AM and traditional methods were added to the discussion section. This will highlight the advantages and limitations of cold spray AM.

3. The κ phase is mentioned in the abstract, but no quantitative evidence (e.g., XRD or EBSD) is provided to verify this feature.

**Answer:** Thank you for your observation. The presence of κ phases was inferred from the XRD pattern (Fig. 6), where characteristic peaks corresponding to κ phases were observed. Although quantitative analysis of phase fractions was not performed, the microstructural refinement visible in optical microscopy and the precipitation of κ phases following heat treatment align with findings reported in literature. Future work should employ EBSD to confirm and quantify the distribution and orientation of κ phases to further validate these observations.
